# Tetramethylpyrazine Attenuates the Endotheliotoxicity and the Mitochondrial Dysfunction by Doxorubicin *via* 14-3-3*γ*/Bcl-2

**DOI:** 10.1155/2019/5820415

**Published:** 2019-12-03

**Authors:** Bin Yang, Hongwei Li, Yang Qiao, Qing Zhou, Shuping Chen, Dong Yin, Huan He, Ming He

**Affiliations:** ^1^Jiangxi Provincial Institute of Hypertension, the First Affiliated Hospital of Nanchang University, Nanchang 330006, China; ^2^Jiangxi Provincial Key Laboratory of Basic Pharmacology, Nanchang University School of Pharmaceutical Science, Nanchang 330006, China; ^3^Jiangxi Provincial Key Laboratory of Molecular Medicine, the Second Affiliated Hospital, Nanchang University, Nanchang 330006, China

## Abstract

Doxorubicin (Dox) with cardiotoxicity and endotheliotoxicity limits its clinical application for cancer. The toxicitic mechanism involves excess ROS generation. 14-3-3s have the protective effects on various injured tissues and cells. Tetramethylpyrazine (TMP) is an alkaloid extracted from the rhizome of Ligusticum wallichii and has multiple bioactivities. We hypothesize that TMP has the protective effects on vascular endothelium by upregulating 14-3-3*γ*. To test the hypothesis, Dox-induced endotheliotoxicity was used to establish vascular endothelium injury models in mice and human umbilical vein endothelial cells. The effects of TMP were assessed by determining thoracic aortic strips' endothelium-dependent dilation (EDD), as well as LDH, CK, caspase-3, SOD, CAT, GSH-Px activities and MDA level in serum, apoptotic rate, and histopathological changes of vascular tissue (*in vivo*). Also, cell viability, LDH and caspase-3 activities, ROS generation, levels of NAD^+^/NADH and GSH/GSSG, MMP, mPTP opening, and apoptotic rate were evaluated (*in vitro*). The expression of 14-3-3*γ* and Bcl-2, as well as phosphorylation of Bad (S112), were determined by Western blot. Our results showed that Dox-induced injury to vascular endothelium was decreased by TMP *via* upregulating 14-3-3*γ* expression in total protein and Bcl-2 expression in mitochondria, activating Bad (S112) phosphorylation, maintaining EDD, reducing LDH, CK, and caspase-3 activities, thereby causing a reduction in apoptotic rate, and histopathological changes of vascular endothelium (*in vivo*). Furthermore, TMP increased cell viability and MMP levels, maintained NAD^+^/NADH, GSH/GSSG balance, decreased LDH and caspase-3 activities, ROS generation, mPTP opening, and apoptotic rate (*in vitro*). However, the protective effects to vascular endothelium of TMP were significantly canceled by pAD/14-3-3*γ*-shRNA, an adenovirus that caused knockdown 14-3-3*γ* expression, or ABT-737, a specific Bcl-2 inhibitor. In conclusion, this study is the first to demonstrate that TMP protects the vascular endothelium against Dox-induced injury via upregulating 14-3-3*γ* expression, promoting translocation of Bcl-2 to the mitochondria, closing mPTP, maintaining MMP, inhibiting RIRR mechanism, suppressing oxidative stress, improving mitochondrial function, and alleviating Dox-induced endotheliotoxicity.

## 1. Introduction

Doxorubicin (Dox) is a broad-spectrum, high efficiency, low cost and convenient use of anticancer antibiotic [1]. However, its dose-dependent cardiotoxicity greatly limits its clinical application [2]. In recent years, the damage of Dox to vascular endothelium, and so-called endotheliotoxicity has also attracted considerable attention [3].

Many studies have found that there are various reasons for Dox's cardiotoxicity or endotheliotoxicity [3, 4]. However, one of the most important reason is that Dox itself may induce oxidative stress, resulting in excessive reactive oxygen species (ROS) generation [3–6]. In previous studies, we have shown that Dox toxicity can cause excessive ROS generation, resulting in severe myocardial damage [7, 8]. However, inhibiting oxidative stress and reducing ROS generation may alleviate cardiotoxicity or endotheliotoxicity induced by Dox [9–13]. Phytochemicals are candidate subjects [7, 8, 10–13].

Tetramethylpyrazine (TMP), an alkaloid extracted from the roots of Ligusticum chuanxiong Hort (LC; Umbelliferae), a traditional Chinese medicine [14], it has multiple targets and many biological functions, such as anti-oxidation, anti-platelet, anti-inflammation, anti-apoptosis and so on [15–17]. Many studies have shown that TMP has protective effects on the myocardium, brain, and vascular endothelium, suggesting that TMP has an excellent application prospect in the prevention and treatment of cardio-cerebrovascular diseases [16–19]. Recently, we have found that TMP could up-regulate 14-3-3*γ* expression, improve mitochondrial function, and reduce apoptosis induced by LPS to cardiomyocytes [20].

14-3-3s is a highly conserved acidic protein family composed of seven isoforms [21]. Through phosphorylation, it interacts with the partner protein and participates in almost all life activities in cells [22]. Our previous studies found that 14-3-3*η* and 14-3-3*γ* participate in acute myocardial injury and protection. 14-3-3*η* participates in ischemia/hypoxia injury and protection, while 14-3-3*γ* mainly involves infection or inflammatory injury and protection [20, 23–29]. Recently, we found that curcumin and quercetin could up-regulate 14-3-3*γ* expression, improve mitochondrial function, and protect the myocardium against Dox's cardiotoxicity [7, 8].

Therefore, the aims of the current study were to investigate by *in vivo* and *in vitro* 1) Whether TMP protected vascular endothelium against endotheliotoxicity induced by Dox; 2) Whether up-regulation of 14-3-3*γ* expression, phosphorylation of Bad (S112) and subsequent translocation of Bcl-2 to the mitochondria were involved in the protection of TMP against endotheliotoxicity induced by Dox; 3) Whether the change of 14-3-3*γ*/Bcl-2 caused by TMP could affect mitochondrial oxidative stress that vascular endothelium induced by Dox endotheliotoxicity; 4) Whether improvement of mitochondrial function mediated by 14-3-3*γ*/Bcl-2 was involved in TMP protecting vascular endothelium against endotheliotoxicity induced by Dox.

## 2. Materials and Methods

### 2.1. Materials, Cells and Animals

Adenovirus pAD/14-3-3*γ*-shRNA and negative control (pAD/scrRNAi) were from GeneChem Co., Ltd (Shanghai, China). TMP, Dox, phenylephrine (PE), sodium nitroprusside (SNP), acetylcholine (Ach), atractyloside (Atr), and ciclosporin A (CsA) were purchased from Sigma-Aldrich (Cat. No. 95162, D1515, P1240000, PHR1423, PHR1546, A6882, C1832, St. Louis, MO, USA). Mitoquinone (MitoQ) was from MedChemExpress (Cat. No. HY-100116, Shanghai, China). ABT-737 was from Selleck (Cat. No. S1002, Houston, TX, USA). Antibodies directed against 14-3-3*γ* was purchased from Santa Cruz (Cat. No. sc-69955, Santa Cruz, CA, USA). Antibodies directed against Bcl-2, Bad phospho-S112, eNOS, eNOS phospho-S1177, cytochrome C (*cyt C*), COX4, and *β*-actin were purchased from Abcam (Cat. No. ab196495, ab129192, ab5589, ab184154, ab16381, ab33985, ab8229, Cambridge, UK). Horseradish peroxidase-conjugated IgG was from Jackson Immuno Research (Cat. No. 107-035-142, West Grove, PA, USA).

Human umbilical vein endothelial cells (HUVECs) were purchased from the China infrastructure of cell line resources (Shanghai, China). Male Kunming mice (8-10 weeks old, weighing 20-22 g) were provided by the Animal Center of Nanchang University (Nanchang, China).

All experimental protocols were performed in accordance with the National Institutes of Health (NIH) Guidelines for the Care and Use of Laboratory Animals (NIH Publication No. 85-23, revised 1996), and approved by the Ethics Committee of Nanchang University (No. 2019-0006).

### 2.2. In Vivo Experiments

Mice were housed (two per cage) in a controlled environment at a temperature of 22°C, humidity of 50%, and a 12-hour light/dark cycle. Water was provided to animals *ad libitum*.

#### 2.2.1. Experimental Grouping In Vivo

**Figure 1:**
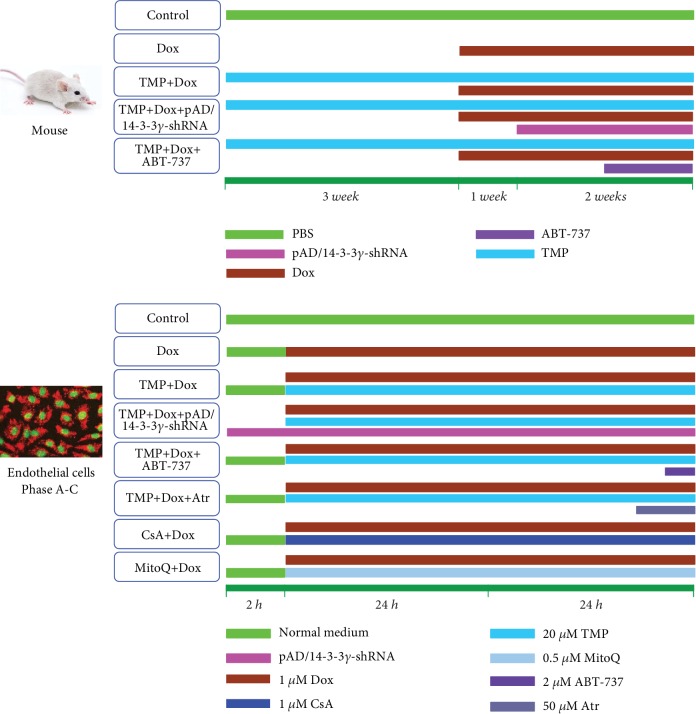
Schematic representation of the experimental design *in vivo* and *in vitro*. *In vivo,* 75 mice were randomly divided into five different groups: Dox group, mice were routinely fed for 3 weeks; then intraperitoneal injected with six injections of 2.5 mg/kg Dox over 3 weeks for a cumulative dose of 15 mg/kg; TMP + Dox group, mice were administered 6 mg/kg TMP, once daily for 6 weeks via intragastric administration, an hour before Dox administration; TMP + Dox + pAD/14-3-3*γ*-shRNA group, mice were treated with a regimen similar to the TMP + Dox group for 4 weeks, then injected with pAD/14-3- 3*γ*-shRNA adenovirus; TMP + Dox + ABT-737 group, mice were treated with a regimen similar to the TMP + Dox group for 5 weeks, followed by a once daily intraperitoneal injection with ABT-737(20 *μ*g/kg), an hour before TMP administration, for 1 week; Control group: mice were given an equal volume of PBS using a regimen similar to the TMP + Dox group. For the *in vitro* experimental groupings: HUVECs in the control group were cultured under normal conditions (37°C, 95% O_2_ and 5% CO_2_) over the entire experiment; HUVECs in the Dox group were treated with 1 *μ*M Dox for 48 hours; HUVECs in the TMP + Dox group were treated similar to the Dox group, but cells were also co-incubated with 20 *μ*M TMP for 48 hours, whereas HUVECs in the TMP + Dox + pAD/14-3-3*γ*-shRNA group were treated with pAD/14-3-3*γ*-shRNA for 2 hours before TMP treatment; HUVECs in the TMP + Dox + ABT-737 group were treated in a manner similar to the TMP + Dox group, but these cells were also co-incubated with 2 *μ*M ABT-737 for an hour; HUVECs in the TMP + Dox + Atr group were treated in a manner similar to HUVECs in the TMP + Dox group, but were also co-incubated for 2 hours with 50 *μ*M Atr; HUVECs in the Dox + CsA/MitoQ groups were treated in a manner similar to the Dox group, but the cells were also co-incubated with 1 *μ*M CsA/0.5 *μ*M MitoQ for 48 hours, respectively. HUVECs that underwent these treatments were combined to form Phase A-C (See the 2.3.2 in the text).

As shown in Figure 1, 75 mice were randomly divided into five groups: the Dox group, mice were routinely fed for 3 weeks; then intraperitoneally injected with six injections of 2.5 mg/kg Dox over 3 weeks for a cumulative dose of 15 mg/kg [7]; the TMP + Dox group, mice were administered 6 mg/kg TMP [30], once daily for 6 weeks via intragastric administration, an hour before Dox administration; the TMP + Dox + pAD/14-3-3*γ*-shRNA group, mice were treated with a regimen similar to the TMP + Dox group for 4 weeks, then injected with pAD/14-3-3*γ*-shRNA adenovirus; the TMP + Dox + ABT-737 group, mice were treated with a regimen similar to the TMP + Dox group for 5 weeks, then followed by a once daily intraperitoneal injection with ABT-737 (20 *μ*g/kg) [31], an hour before TMP administration, for 1 week; and the Control group, mice were given an equal volume of phosphate buffered saline (PBS) using a regimen similar to the TMP + Dox group.

#### 2.2.2. Gene Delivery via Tail Vein

The 14-3-3*γ* knockdown model was constructed in Kunming mice *via* tail vein injection of a recombinant adenovirus containing the shRNA of 14-3-3*γ* gene (Genbank ID 22628, target sequence: GCTTCTGAGGCAGC GTATA) as previously described [32]. Briefly, pAD/14-3-3*γ*-shRNA adenovirus (2 × 10^11^ plaque-forming units/ml, 200 *μ*l) was injected into the tail vein. Two weeks post-injection, mice were killed.

#### 2.2.3. Collection of Blood and Tissue

At the end of the experiment, mice were anesthetized using an intraperitoneal injection with ketamine (100 mg/kg) and xylazine (8 mg/kg). Then, blood was collected in heparinized capillary tubes via a cardiac puncture and immediately centrifuged for 10 min at 3000 rpm and 25°C for serum separation. Thoracic aorta rings were harvested in ice-cold physiologic saline solution (PSS: 0.288 g NaH_2_PO_4_, 1.802 g glucose, 0.44 g sodium pyruvate, 20.0 g BSA, 21.48 g NaCl, 0.875 g KCl, 0.7195 g MgSO_4_ 7H_2_0, 13.9 g MOPS sodium salt, and 0.185 g EDTA per liter solution at pH 7.4) and evaluated for vascular reactivity as described previously [33].

#### 2.2.4. Determination of Biochemical and Tissues Injury Indexes

As a biomarker of tissue injury, the activities of serum lactate dehydrogenase (LDH), and creatine kinase (CK) were measured by a microplate reader (Bio-rad 680, Hercules, CA, USA) according to the specifications of their respective assay kit (Nanjing Jiancheng Bioengineering Institute Co. Ltd., Nanjing, China).

The ferric reducing antioxidant power (FRAP) assay (Cell Biolabs, Inc. Santiago, CA, USA), which is based on reduction of the ferric tripyridyltriazine (Fe^3+^-TPTZ) complex to ferrous (Fe^2+^-TPTZ), was used to evaluate the antioxidant potential of the serum of mice at an absorption of 560 nm [29, 34]. In brief, 5 *μ*l serum and 15 *μ*l deionized water were mixed with 75 *μ*l FRAP color solution. The mixture was incubated for 30 min at 25°C and its OD was measured at a wavelength of 560 nm by a spectrophotometer. A ferrous chloride standard was used to prepare the standard curve. The concentration of samples, indicating antioxidant potential, was obtained using the equation of the standard curve.

Malondialdehyde (MDA), superoxide dismutase (SOD), catalase (CAT), and glutathione peroxidase (GSH-Px) are vital indexes for estimating oxidative stress [29, 35]. The antioxidant enzyme activities and lipid peroxidation levels in mice serum were determined according to the manufacturer's instructions. In brief, collected supernatants were measured using a microplate reader. Kits for measuring MDA level, SOD, CAT, and GSH-Px activities were purchased from Nanjing Jiancheng Bioenginering Institute Co. Ltd. (Nanjing, China).

#### 2.2.5. Hematoxylin–Eosin (H&E) Staining and the Terminal Deoxynucleotidyl Transferase-Mediated Nick End Labeling (TUNEL) Assay

Freshly harvested thoracic aortas were fixed in 10% buffered formalin solution embedded in paraffin and sectioned into 5 *μ*m thick sections that were mounted onto glass slides. To evaluate morphological changes, H&E staining was performed. In addition, to detect apoptosis, TUNEL (Promega, Madison, WI, USA) staining method was performed according to the manufacturer's guidelines [7].

#### 2.2.6. Vascular Reactivity

Vascular contractility and relaxation were determined as previously described [33, 36]. Briefly, thoracic aortas were placed in pressure myograph chambers (DMT Inc., Atlanta, GA, USA), containing warm PSS, cannulated and secured onto glass micropipettes, and equilibrated at an intraluminal pressure of 50 mmHg for an hour at 37°C. First, we confirmed that arteries maintained constriction to phenylephrine (PE: 10^−10^-10^−4^ M) for the duration of the experiment until no spontaneous dilatation occurred during the constriction period (i.e. 5-12 min). Then, the samples were constricted by increasing doses of PE (10^−6^ M, about EC_50_), immediately followed by a dose-response with endothelium-dependent dilator acetylcholine (ACh: 10^−9^-10^−4^ M). After a washout period and after pre-constriction to PE (10^−6^ M), a dose-response to the endothelium-independent dilator sodium nitroprusside (SNP: 10^−10^-10^−4^ M) was performed. The percent of dilation was calculated based on the maximal luminal diameter of each artery.

#### 2.2.7. Determination of Nitric Oxide (NO) Contents

The NO content in mice's serum or the culture medium was indirectly reflected by the contents of nitrite and nitrate [37]. Nitrate is converted to nitrite by aspergillus nitrite reductase, and the total level of nitrite was measured using the Griess reagent (G4410, Sigma-Aldrich), for which the absorbance was determined at 540 nm. The NO content in samples was presented as the amounts of nitrite and nitrate (*μ*M) per gram protein of serum or per liter of culture medium.

#### 2.2.8. Western Blot Analysis

The total amount of protein from thoracic aortas samples, as well as total amount of protein and the mitochondrial proteins from HUVECs, were extracted using a protein extraction kit (Applygen Technologies Inc, Beijing, China), respectively. The protein concentration was determined by Bradford method. A total of 50 *μ*g of protein was separated by denaturing SDS-polyacrylamide gel electrophoresis and transferred to polyvinylidene fluoride membranes. Membranes were then blocked with 5% skim milk, washed, incubated with primary antibodies directed against 14-3-3*γ* (1 : 1000), Bcl-2 (1 : 500), Bad phospho-S112 (1 : 500), eNOS (1 : 1000), eNOS phospho-S1177 (1 : 1000), *cyt C* (1 : 1000), *β*-actin (1 : 2000), and COX4 (1 : 1000), then incubated with Horseradish peroxidase-conjugated secondary antibody. Subsequently, membranes were incubated with an enhanced chemiluminescence reagent for 2 min at room temperature, and protein bands were visualized using an enhanced chemiluminescence method and analyzed with Quantity One software (Bio-Rad, Hercules, CA, USA) [7].

### 2.3. Experiments In Vitro

#### 2.3.1. Endothelial Cell Culture and Adenovirus Transfection

For transfection assays, HUVECs were cultivated in high-glucose Dulbecco's modified Eagle medium (DMEM, Gibco-BRL, Grand Island, NY, USA) supplemented with 10% heat- inactivated fetal bovine serum (FBS, Gibco-BRL), penicillin (100 U/mL), and streptomycin (100 *μ*g/mL) and cultured at 37°C in a humidified atmosphere at 5% CO_2_.

Adenovirus pAD/14-3-3*γ*-shRNA and negative control-pAD/scrRNAi (shRNA of 14-3-3*γ* gene, the target sequence: GCTTCTGAGGCAGCGTATA and the negative control sequence: TTCTCCGAACGTGTCACGT) were transfected into HUVECs cultured in fresh DMEM supplemented with 15% FBS. Transfection efficiency was roughly 85% after 48 hours. Transfected cells were incubated at 37°C, 95% O_2_, and 5% CO_2_ for 2 hours before use in the experiments.

#### 2.3.2. Experimental Design (Figure 1)

*(1) Phase A.* Firstly, we investigated whether TMP-treated HUVECs confirmed protective effects against the endotheliotoxicity induced by Dox. As well, the optimal concentration of TMP-treated was determined.

Cells were randomly divided into the following experimental groups: HUVECs in the control group were cultured under normal conditions (37°C, 95% O_2_ and 5% CO_2_) over the entire experiment; HUVECs in the Dox group were treated with 1 *μ*M Dox for 48 hours [7]; HUVECs in the TMP + Dox group were treated similar to the Dox group, but cells were also co-incubated with different concentrations of TMP (10, 20, 40, or 80 *μ*M) for 48 hours. At the end of the experiments, cell viability and LDH activity were determined.

*(2) Phase B*. Next, we further confirmed whether 14-3-3*γ* expression and Bcl-2 activity could affect the protective effects of TMP on Dox's endotheliotoxicity.

Cells were randomly divided into five groups. Thereinto, the control, and the Dox group were treated with the above (1). HUVECs in the TMP + Dox group were treated similar to the Dox group, but cells were also co-incubated with 20 *μ*M TMP for 48 hours, whereas cells in the TMP + Dox + pAD/14-3-3*γ*-shRNA groups were treated with pAD/14-3-3*γ*-shRNA for 2 hours before TMP treatment; HUVECs in the TMP + Dox + ABT-737 group were treated similar to the TMP + Dox group, but these cells were also co-incubated with 2 *μ*M ABT-737 for an hour [31]. At the end of the experiments, cell viability, apoptosis, LDH and caspase 3 activities, NO levels in the culture medium, the expression of 14-3-3*γ*, Bad phospho-S112, eNOS, and eNOS phospho-S1177 in the lysate of HUVECs, and Bcl-2 expression in mitochondria were determined.

*(3) Phase C*. Finally, we investigated how TMP-treated HUVECs maintain mitochondrial function and possible mechanisms.

In brief, HUVECs were randomly divided into six groups. Thereinto, HUVECs in the control, the Dox, and the TMP + Dox group were treated with the above (2). HUVECs in the TMP + Dox + Atr group were treated similar to cells in the TMP + Dox group, but were also co-incubated for 2 hours with 50 *μ*M Atr [38]; HUVECs in the Dox + CsA/MitoQ groups were treated similar to the Dox group, but the cells were also co-incubated with 1 *μ*M CsA/0.5 *μ*M MitoQ for 48 hours, respectively [39, 40].

At the end of the experiments, cell viability and LDH activity, oxygen consumption rate (OCR), extracellular acidification rate (ECAR), intracellular and mitochondrial ROS generation, levels of NAD^+^, NADH, GSH, and GSSG in mitochondria/intracellular, mitochondrial membrane potential (MMP), mitochondria permeability transition pore (mPTP) opening, and *cyt C* release from mitochondria to cytoplasm in HUVECs were determined.

#### 2.3.3. 3-(4,5-Dimethylthiazol-2-Yl)-5-(3-Carboxymethoxyphenyl)-2-(4-Sulfophenyl)-2H -Tetrazolium (MTS) Assay

Cells were plated in 96-well plates at a density of 1 × 10^4^ cells/well, incubated at 37°C with 20 *μ*l MTS (5 mg/ml, Promega, Madison, WI, USA) in 100 *μ*l of DMEM medium for 2 hours. Next, the absorbance of each well was measured at 490 nm by a microplate reader (Bio-Rad680). The absorbance was directly proportional to the number of live cells.

#### 2.3.4. Measurement of LDH and Caspase-3 Activities

In HUVECs, LDH is an intracellular enzyme that is released into the culture medium upon cell damage [7]. In this study, at the end of the experiment, the supernatant was collected, and the LDH activity was determined by a microplate reader (Bio-rad 680) according to the specifications of the LDH assay kit (Nanjing Jiancheng Bioengineering Institute Co. Ltd.).

Caspase-3 activity was measured in the cytosolic fraction of isolated HUVECs as described previously [7]. Briefly, caspase-3 activity was determined by measuring the cleavage of a caspase-3-specific substrate [acetyl-Asp-Glu-Val-Asp(DEVD)-p-nitroanilide (pNA)(DEVD-pNA)] using a caspase-3 activity assay kit (R&D Systems, Minneapolis, MN, USA) according to the manufacturer's instructions.

#### 2.3.5. Assessment of Endothelial Apoptosis Using Annexin V-FITC and PI

Assessment of apoptosis of HUVECs was performed using an Annexin V-EGFP/PI apoptosis detection kit (BD Biosciences, San Diego, CA, USA). Annexin V-stained cells were analyzed using a Cytomics FC500 flow cytometer (Beckman Coulter, Brea, CA, USA) and DCF fluorescence was determined, which is an index of cellular damage [7].

#### 2.3.6. Measurement of Intracellular and Mitochondrial ROS

Intracellular and mitochondrial ROS generation was measured using a DCFH-DA or mitoSOX probe as previously method [41]. In brief, cells were harvested and washed with serum-free DMEM media. Then, cells were mixed with serum-free media containing 10 *μ*M DCFH-DA probe (Molecular Probes, Eugene, OR, USA) or 5 *μ*M mitoSOX probe (Thermo Fisher Scientific, Waltham, MA, USA) and incubated at 37°C in the dark for 30 min with slight agitation every 5 min. Subsequently, cell pellets were collected, washed three times with PBS, and resuspended in 500 *μ*L PBS for flow cytometry analysis (Cytomics FC500). The induced green fluorescence from 10,000 cells was documented at 488 or 510 nm. Flow Jo software was used to analyze the average fluorescence intensity.

#### 2.3.7. Evaluation of OCR and ECAR

Mitochondrial respiration is an indicator of the functional bioenergetics capacity of the mitochondria and overall cellular health [29, 42]. In the present study, we used an XFp Extracellular Flux Analyzer (Seahorse Biosciences, North Billerica, MA, USA) to evaluate the OCR, which was measured as a function of time. In brief, HUVECs were seeded in Seahorse XFp cell cultured miniplates at a density of 5,000 cells/well and subjected to the corresponding treatment. After analysis of basal respiration, oligomycin (complex V inhibitor, 10 *μ*M), carbonyl-cyanide-4-(trifluoromethoxy) phenylhydrazone (FCCP, permeabilizes the inner mitochondrial membrane for protons, 2 *μ*M), rotenone/antimycin A (inhibitors of complex I and III, 0.5 *μ*M/0.5 *μ*M) were added sequentially. Wells without cells served as background and were used to normalize the reading of each well to the background noise of the plate. OCR was normalized to total protein per well.

To monitor glycolytic function, ECAR, expressed as mpH/min, was determined. The measurement procedure was similar to that of OCR described above. After measurement of basal ECAR, glucose solution (80 mM), oligomycin (5 mM), and 2-DG (100 mM) were added sequentially to determine glycolysis, glycolytic capacity, and the glycolytic reserve, respectively [42].

#### 2.3.8. Isolation of Mitochondrial Fractions and Assessment of Nicotinamide Adenine Dinucleotide Mitochondrial

At the end of the experiments, HUVECs were collected and washed twice with cold PBS. The cells were resuspended in 1 × Cytosol Extraction Buffer Mix and homogenized on ice. The mixture was left on ice for 15 min and centrifuged at 1,300 × g for 5 min at 4°C. The supernatant was then transferred into a new tube and spun at 17,000 × g for 10 min at 4°C to precipitate the mitochondria. This pellet was resuspended in 15% Percoll (Cat. No. P4937, Sigma- Aldrich), and layered onto a preformed gradient of 22% Percoll, then layered onto 50% Percoll. The Percoll density gradient was centrifuged at 17,000 × g for 10 min at 4°C, and purified mitochondria were collected at the interface between the 50% and 22% gradients. The purified mitochondrial sample was centrifuged at 7,000 × g for 10 min at 4°C [27, 43].

Nicotinamide adenine dinucleotide exists in an oxidized (NAD^+^) form and a reduced (NADH) form. Total NAD^+^ and NADH levels in the purified mitochondrial samples were quantified using an NAD^+^/NADH quantification kit according to the manufacturer's instructions (Biovision, Milpitas, CA, USA) [43]. Total NAD^+^/NADH levels were assessed by converting all NAD^+^ in the samples to NADH using the NAD Cycling Enzyme Mix and NADH Developer included in the kit. Nicotinamide adenine dinucleotide reduced-only concentrations were achieved by heating the mitochondrial samples to 60°C for 30 min to decompose NAD^+^. Reduced nicotinamide adenine dinucleotide was measured at OD 450 nm using a spectrophotometer microplate reader (BioTek Instruments, Winooski, VT, USA). Known concentrations of purified NADH were used to generate standard curves and to calculate mitochondrial concentration. Values of NAD^+^ were generated by subtracting the NADH-only value from the total NAD^+^/NADH value.

#### 2.3.9. Assessment of Intracellular GSH, GSSG, and GSH/GSSG Ratio

Reduced glutathione is the most abundant intracellular thiol. The intracellular redox state is reflected by the levels of oxidized (GSSG) and reduced (GSH) glutathione. The GSH/GSSG ratio is also an essential indicator of cellular health [44, 45]. Measurements were performed according to the manufacturer's instructions using supernatants from cells lysed after treatment. GSH, GSSG, and GSH/GSSG ratio assay kits were purchased from Beyotime Institute of Biotechnology (Haimen, Jiangsu, China).

#### 2.3.10. Assessment of MMP

Flow cytometry analysis was used to assess the loss of MMP using the fluorescent indicator, JC-1 (5, 5', 6, 6'-tetrachloro-1,1',3,3'-tetraethyl-benzimidazole carbocyanine iodide, Invitrogen, Carlsbad, CA, USA). HUVECs were harvested and the cell suspension was incubated with JC-1 (200 *μ*M) at 37°C for 20 min followed by two rounds of washing with PBS to remove the remaining reagents. Fluorescence was measured with a Cytomics FC500 flow cytometer with initial excitation and emission wavelengths (ex/em) of 530 and 580 nm (red), followed by ex/em at 485/530 nm (green). The ratio of red to green fluorescence intensity of cells indicated the level of MMP [7].

#### 2.3.11. Opening of mPTP

In cell apoptosis, mPTP opening plays a major role and the Ca^2+^ induced mitochondria swelling assay can be used to determine mPTP opening. The isolated mitochondria were resuspended in swelling buffer (KCl 120 mM, Tris-HCl 10 mM, MOPS 20 mM, KH_2_PO_4_ 5 mM), and poured into a 96-well microtiter plate. The addition of 40 *μ*l of CaCl_2_ solution (200 nM) to each well served as a stimulant of mPTP opening and resulted in a steady decline in mitochondrial density. The absorbance at 520 nm was measured every minute until stable values were observed. To measure the extent of mPTP opening, changes in absorbance were calculated [7].

### 2.4. Statistical Analysis

All values were expressed as the means ± standard error of mean (SEM). One-way analysis of variance (ANOVA) was employed to test the significance of differences in the biochemical data across groups, followed by post hoc testing for individual differences. The results were considered significant at a value of *P* < 0.05.

## 3. Results

### 3.1. TMP-Treated Alleviates Vascular tissue's Damage Induced by Dox Toxicity in Mice

**Figure 2:**
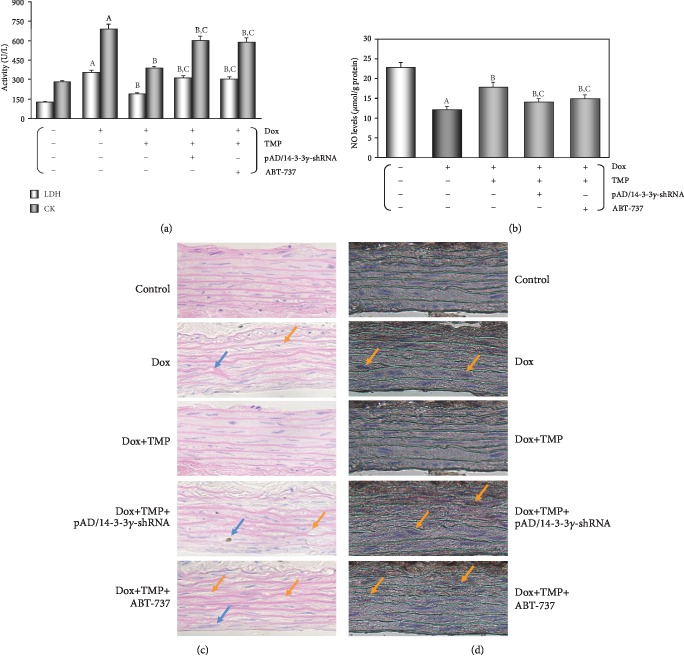
TMP-treated alleviates vascular tissue's damage induced by Dox toxicity in mice. (a) The activities of mice's serum LDH and CK. (b) Serum contents of NO. (c) H&E staining was performed for morphological analysis in the thoracic aortas tissue. Blue arrow: spotty necrosis; Orange arrow: hypertrophy of interstitial cells. (d) TUNEL staining was performed for morphological analysis in the thoracic aortas tissue. Orange arrow: TUNEL-positive cells. Data are presented as the mean ± SEM. for fifteen individual experiments. a: *P* < 0.01, versus control group; b: *P* < 0.01, versus Dox group; c: *P* < 0.01, versus TMP + Dox group.

As shown in Figure 2(a), as expected, the activities of serum LDH and CK in the Dox group were significantly increased (*P* < 0.01), which were significantly decreased following TMP-treated (*P* < 0.01), and restored mostly when combined with pAD/14-3-3*γ*-shRNA or ABT-737 (*P* < 0.01), indicating that Dox had caused tissue and/or organ damage in mice, TMP could effectively resist it, but which depended on 14-3-3*γ* expression and Bcl-2 activity.

Histopathological examination of mice's thoracic aortas also confirmed the protective effects of TMP on Dox-induced vascular toxicity. As shown in Figure 2(c), in the Dox's mice, some inflammatory changes, such as inflammatory infiltration, and cell swelling, were found in the thoracic aorta's tissue. However, tissue injury was significantly reduced with TMP-treated. Further, the apoptosis of thoracic aorta's tissue was assayed using TUNEL staining (Figure 2(d)). In microscopy, the Dox group clearly promoted apoptosis of the thoracic aorta's tissue, which was significantly reversed by TMP-treated. However, when combined with pAD/14-3-3*γ*-shRNA or ABT-737, the above protective effects of TMP were mostly canceled.

**Figure 3:**
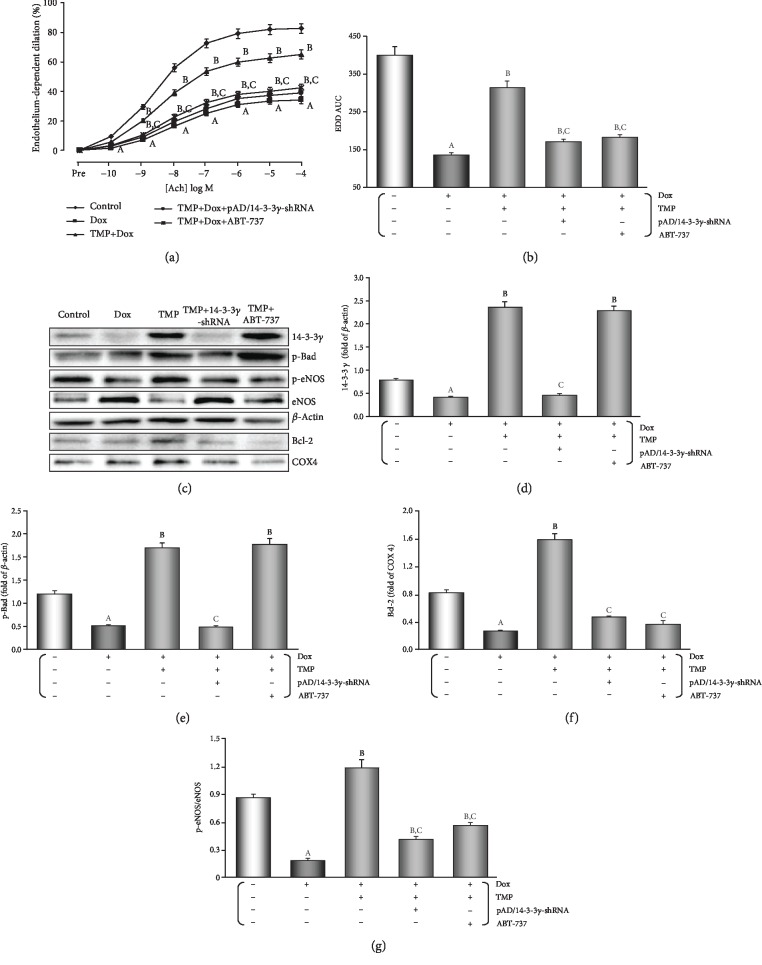
TMP-treated protects VECs' function against the endotheliotoxicity by Dox in mice. (a) Endothelium-dependent dilation (EDD) of the thoracic aortic strips. (b) Area under of the curve for EDD of the thoracic aortic strips. (c) Western blot's banding of related proteins in the aortic tissue. (d) Histogram of 14-3-3*γ* expression in the cytoplasm. (e) Histogram of p-Bad expression in the cytoplasm. (f) Histogram of Bcl-2 expression in the mitochondria. (g) Histogram of p-eNOS/eNOS expression in the cytoplasm. On (c), from left to right, lane 1: control; lane 2: Dox; lane 3: TMP + Dox; lane 4: TMP + Dox + pAD/14-3-3*γ*-shRNA; lane 5: TMP + Dox + ABT-737. Data are presented as the mean ± SEM. for fifteen individual experiments. a: *P* < 0.01, versus control group; b: *P* < 0.01, versus Dox group; c: *P* < 0.01, versus TMP + Dox group.

As illustrated in Figure 2(b), the serum contents of NO in the Dox's mice were much lower (*P* < 0.01), which were significantly reversed by TMP-treated (*P* < 0.01). This change could be mostly completely counteracted by pAD/14-3-3*γ*-shRNA or ABT-737 (*P* < 0.01). Furthermore, we detected the expression of eNOS and p-eNOS of the thoracic aorta's tissue in all mice, respectively. As illustrated in Figures 3(c) and 3(g), the aortic tissue in the Dox's mice, the p-eNOS/eNOS ratio was reduced (*P* < 0.01), which significantly reversed by TMP-treated (*P* < 0.01), but it could be only partly counteracted by pAD/14-3-3*γ*-shRNA or ABT-737 (*P* < 0.01). The above results indicated that TMP-treated could promote the phosphorylation of eNOS in the aortic tissue, increase NO synthesis, which also only partly depended on 14-3-3*γ* expression and Bcl-2 activity.

### 3.2. TMP-Treated Protects the Vascular endothelia's Function against the Endotheliotoxicity by Dox in Mice

Generally, the control experiments of endothelium-dependent dilation (EDD) with Ach and endothelium-independent dilation (EID) with SNP are noteworthy criteria for judging whether the vascular endothelium's function normally or not [33]. As shown in Figures 3(a) and 3(b), EDD in the Dox group was markedly impaired (*P* < 0.01), and area under the curve (AUC) of dose-effect relationship decreased to 34.3% of the control group (*P* < 0.01). TMP-treated improved EDD such that dilation was significantly increased at several doses of Ach (*P* < 0.01), and AUC also recovered to 78.6% of the control group (*P* < 0.01), but it could be mostly also reversed by pAD/14-3-3*γ*-shRNA or ABT-737 to 42.5% and 45.5% of the control group, respectively (*P* < 0.01). Similarly, EID in the Dox group was significantly impaired, and the AUC was 28.3% compared to the control group, TMP-treated improved could also reverse the related changes (*P* < 0.01, [Supplementary-material supplementary-material-1] of the section of Supplementary materials). This indicated that TMP-treated could significantly alleviate vascular endothelium's damage by Dox toxicity, it also has considerable to ease harm to vascular smooth muscle, of course, which also depended on 14-3-3*γ* expression and Bcl-2 activity.

As illustrated in Figures 3(c)–3(f), it was interesting that in the aortic tissue TMP-treated up-regulated 14-3-3*γ* expression and promoted the phosphorylation of Bad, especially upregulation of mitochondrial Bcl-2 expression (*P* < 0.01). However, ABT-737, a specific Bcl-2 inhibitor [31], did not affect up-regulation of 14-3-3*γ* expression and Bad phosphorylation by TMP-treated (*P* > 0.05), but could significantly reduce Bcl-2 expression by TMP-treated in mitochondria (*P* < 0.01).

### 3.3. TMP-Treated Protects HUVECs against the Endotheliotoxicity Induced by Dox

**Figure 4:**
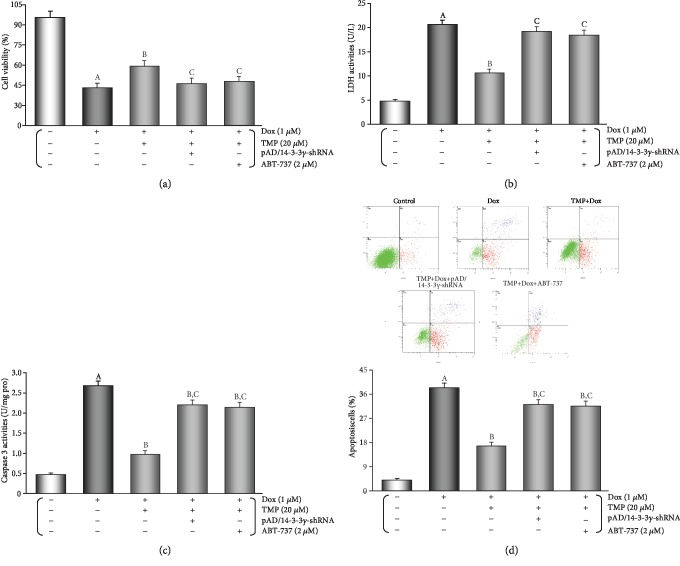
TMP-treated protects HUVECs against the endotheliotoxicity induced by Dox. (a) Cell viability of HUVECs. (b) LDH activities in culture media. (c) Histogram of the caspase-3 activity in HUVECs. (d) Flow cytometry dot plots (*x*-axis: annexin V-staining, *y*-axis: PI staining), and the quantitation of apoptotic cells. Data are presented as the mean ± SEM. for eight individual experiments. a: *P* < 0.01, versus control group; b: *P* < 0.01, versus Dox group; c: *P* < 0.01, versus TMP + Dox group.

Cell viability and LDH leakage generally serve as indexes of cell injury [7]. To evaluate the effects of TMP-treated, we tested different concentrations of TMP-treated on HUVECs that were subjected to Dox toxicity. As shown in [Supplementary-material supplementary-material-1] of the section of Supplementary materials, HUVECs subjected to Dox toxicity showed a decrease in cell viability (*P* < 0.01) and an increase in LDH activity (*P* < 0.01). TMP-treated significantly increased cell viability and reduced LDH activity (*P* < 0.01) in a concentration-dependent manner. The optimal concentration of TMP-treated was determined to be 20 *μ*M by chosen for subsequent experiments. Interestingly, co-treatment by pAD/14-3-3*γ*-shRNA or ABT-737 mostly abolished the protective effects of TMP-treated (Figures 4(a) and 4(b), *P* < 0.01).

When compared with the control group, cell viability and LDH activity did not change when using TMP alone, CsA alone, MitoQ alone, pAD/scrRNAi alone, TMP+ pAD/14-3-3*γ*-shRNA, TMP + pAD/scrRNAi, TMP + ABT-737, and TMP + Atr (*P* > 0.05), however, treatment with pAD/14-3-3*γ*-shRNA alone, ABT-737 alone or Atr alone, cell viability was lower and LDH activity was higher compared to that of the control group (*P* < 0.01, [Supplementary-material supplementary-material-1] of the section of Supplementary materials), indicating that 14-3-3*γ* expression, Bcl-2 activity, and mPTP closing play a vital role in maintaining normal HUVECs function. This was also the case for the pAD/14-3-3*γ*-shRNA+Dox, the ABT-737 + Dox, and the Atr + Dox groups compared with the Dox group (*P* < 0.01). But cell viability and LDH activity did not change when using pAD/scrRNAi+Dox (*P* > 0.05, [Supplementary-material supplementary-material-1] of the section of Supplementary materials), indicating that could aggravate HUVECs injury using pAD/14-3-3*γ*-shRNA downregulates 14-3-3*γ* expression, or ABT-737 inhibits Bcl-2, or Atr opens the mPTP. However, pAD/scrRNAi as a negative control could not affect cell viability and LDH activity.

As shown in Figure 4(c), the caspase-3 activity in the Dox group was significantly increased when compared to that in the control group (*P* < 0.01), whereas TMP-treated significantly inhibited the caspase-3 activity when compared to the Dox group (*P* < 0.01). The caspase-3 activities of increased upon TMP plus pAD/14-3-3*γ*-shRNA or ABT- 737 (*P* < 0.01).

The degree of HUVECs apoptosis was evaluated using Annexin V/PI double staining, then analyzed by flow cytometry (Figure 4(d)). The results showed that when compared with the Dox group, apoptosis by TMP-treated was significantly reduced (*P* < 0.01). However, a higher rate of apoptosis was observed in cells with TMP plus pAD/14-3-3*γ*-shRNA or ABT-737 (*P* < 0.01).

### 3.4. TMP-Treated Alters the Expression, Phosphorylation, or Sublocalization, or Metabolism of the Related Active Proteins in HUVECs of Dox Injury

**Figure 5:**
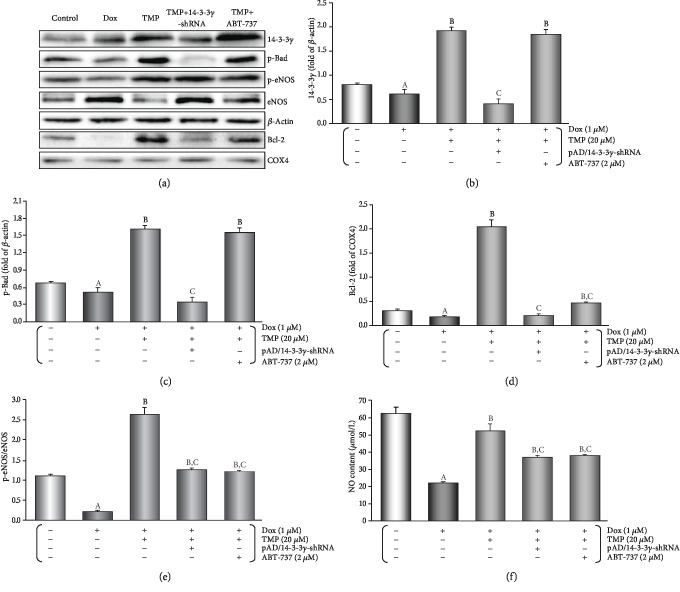
TMP-treated alters the expression, phosphorylation, or sublocalization, or metabolism of the related active proteins in HUVECs of Dox injury. (a) Western blot's banding of the related proteins in HUVECs. (b) Histogram of 14-3-3*γ* expression in the cytoplasm. (c) Histogram of p-Bad expression in the cytoplasm. (d) Histogram of Bcl-2 expression in the mitochondria. (e) Histogram of p-eNOS/eNOS expression in the cytoplasm. (f) The contents of NO in culture medium. On (a), from left to right, lane 1: control; lane 2: Dox; lane 3: TMP + Dox; lane 4: TMP + Dox + pAD/14-3-3*γ*-shRNA; lane 5: TMP + Dox + ABT-737. Data are presented as the mean ± SEM. for eight individual experiments. a: *P* < 0.01, versus control group; b: *P* < 0.01, versus Dox group; c: *P* < 0.01, versus TMP + Dox group.

As illustrated in Figures 5(a)-5(d), in HUVECs TMP-treated significantly up-regulated 14-3-3*γ* expression and promoted the phosphorylation of Bad, especially upregulation of mitochondrial Bcl-2 expression (*P* < 0.01), TMP plus pAD/14-3-3*γ*-shRNA could completely reverse the above changes. However, ABT-737 had no effect on up-regulation of 14-3-3*γ* expression and Bad phosphorylation by TMP (*P* > 0.05), but could significantly reduce Bcl-2 expression in mitochondria caused by TMP-treated (*P* < 0.01).

When compared with the control group, 14-3-3*γ* expression did not change when using pAD/scrRNAi alone-treated, as same as using TMP + pAD/scrRNAi+Dox and TMP + Dox (*P* > 0.05, [Supplementary-material supplementary-material-1] of the section of Supplementary materials), indicating that pAD/scrRNAi did not affect 14-3-3*γ* expression as a negative control.

As illustrated in Figures 5(a), 5(e) and 5(f), in HUVECs, the p-eNOS/eNOS ratio increased significantly (*P* < 0.01) after TMP-treated, and NO content increased significantly (*P* < 0.01) as a follow-up result, but pAD/14-3-3*γ*-shRNA or ABT-737 could partly counteract it (*P* < 0.01). The above results indicated that TMP-treated could promote the phosphorylation of eNOS in HUVECs, increase NO synthesis, which also only partly depended on 14-3-3*γ* expression and Bcl-2 activity.

### 3.5. TMP-Treated Preserves the Intracellular/Mitochondrial Balance between ROS Generation and Their Neutralisation in HUVECs

It has previously been shown that oxidative stress plays a key role in Dox toxic injury [7, 8]. First, we found that HUVECs were treated by 1 *μ*M Dox added 1 *μ*M CsA [39], a mPTP closing agent, or 0.5 *μ*M MitoQ [40], a mitochondria-targeted CoQ-10 antioxidant co-incubation, cell viability increased and LDH activity in the culture medium decreased, these results indicated that CsA and MitoQ alleviate caused HUVECs injury by Dox toxicity. However, 50 *μ*M Atr, a potent mPTP opener, could completely reverse the protective effects of 20 *μ*M TMP, that is, it could decrease cell viability and increase LDH activity in the culture medium (*P* < 0.01, [Supplementary-material supplementary-material-1] of the section of Supplementary materials).

**Figure 6:**
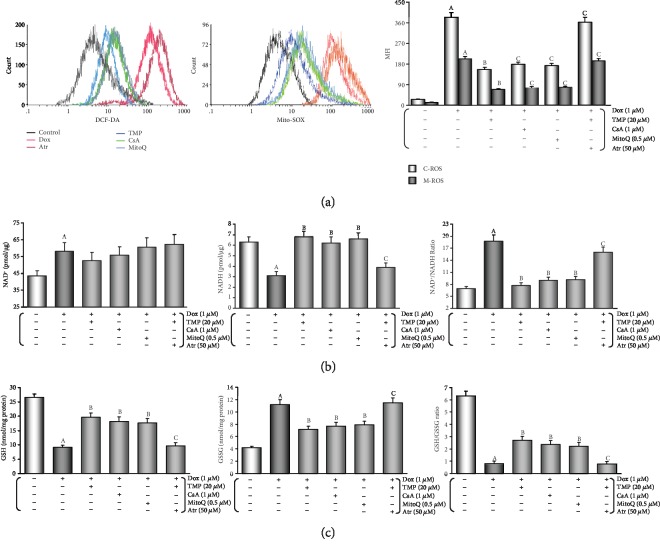
TMP-treated preserves the intracellular/mitochondrial balance between ROS generation and their neutralisation in HUVECs. (a) Intracellular/mitochondrial ROS generation of HUVECs after different treatments. Left: Intracellular ROS generation; middle: mitochondrial ROS generation; right: histogram of the intracellular/mitochondrial ROS generation at various treatments. MFI: mean fluorescence intensity. C-ROS: intracellular ROS. M-ROS: mitochondrial ROS. (b) mitochondrial nicotinamide adenine dinucleotide levels of HUVECs after different treatments. Left: histogram of mitochondrial NAD^+^ levels at various treatments; middle: histogram of mitochondrial NADH levels at various treatments; right: histogram of mitochondrial NAD^+^/NADH ratio at various treatments. (c) Intracellular glutathione levels of HUVECs after different treatments. left: histogram of intracellular GSH levels at various treatments; middle: histogram of intracellular GSSG levels at various treatments; right: histogram of intracellular GSH/GSSG ratio at various treatments. Data are presented as the mean ± SEM for eight individual experiments. a: *P* < 0.01, versus control group; b: *P* < 0.01, versus Dox group; c: *P* < 0.01, versus TMP + Dox group.

After adding Dox for 48 hours, the peak of intracellular/mitochondrial ROS in HUVECs was significantly moved to the right, indicating both significant increase in intracellular/mitochondrial ROS generation of the Dox group (*P* < 0.01, Figure 6(a)). Moreover, adding TMP/CsA/MitoQ co-incubation caused a significant shift of the peak of intracellular/mitochondrial ROS in HUVECs to the left, which indicated a significant decrease in intracellular/mitochondrial ROS generation when compared with the Dox group (*P* < 0.01). However, adding Atr co-incubation could reverse TMP's effect (*P* < 0.01), this strongly suggests that mPTP is open or closed and plays an important role in intracellular/mitochondrial ROS generation.

Several small molecular markers such as NAD^+^/NADH [43, 45] and GSH/GSSG [44, 46] in mitochondria/intracellular are useful indexes of redox state and redox balance [47]. In HUVECs, the content of NADH and GSH, GSH/GSSG ratio increased significantly (*P* < 0.01) and the content of NAD^+^ and GSSG, NAD^+^/NADH ratio decreased (*P* < 0.01) in TMP treatment (Figures 6(b) and 6(c)); these results were similar to that achieved with CsA and MitoQ treatment. This results further confirm that TMP, like CsA or MitoQ, could significantly reduce oxidative stress and ROS generation in mitochondria. The effects were also found to directly relate to mitochondrial mPTP opening as Atr, a potent mPTP opener, could almost completely cancel the effect of 20 *μ*M TMP (*P* < 0.01). This phenomenon was consistent with the changes in cell viability and LDH activity mentioned above.

Usually, in animal serum, MDA content, SOD, CAT, and GSH-Px activities are determined to assess the level of oxidative stress [7, 29], and FRAP assay is used to evaluate the antioxidant potential [29, 34]. In this study, as shown in Table 1, in mice's serum after TMP-treated, SOD, CAT, and GSH-Px activities were significantly increased and MDA content was decreased (*P* < 0.01). Consistent with the lower level of oxidative stress, the FRAP results indicated a high antioxidant potential in the TMP group (*P* < 0.01), however, the above protective effects of TMP-treated could be mostly counteracted by pAD/14-3-3*γ*-shRNA or ABT-737 (*P* < 0.01).

### 3.6. TMP-Treated Maintains Mitochondrial Function in HUVECs

**Figure 7:**
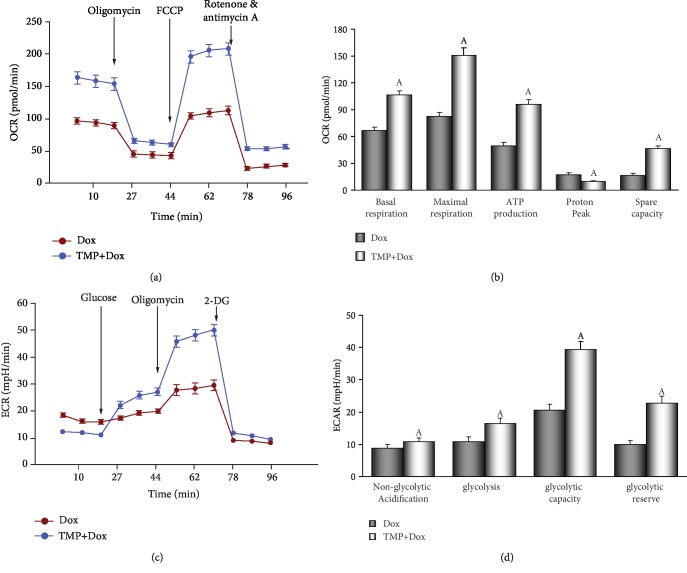
TMP-treated defends mitochondrial respiration and metabolize in HUVECs. (a) Mitochondrial oxygen consumption rate (OCR) curves obtained from different treatme. (b) Histogram of OCR important parameter from different treatme. (c) Mitochondrial extracellular acidification rate (ECAR) curves obtained from different treatme. (d) Histogram of ECAR important parameter from different treatme. Data are presented as the mean ± SEM. for three individual experiments. a: *P* < 0.01, versus Dox group.

To explore the protective effects of TMP on mitochondrial respiration, OCR was measured using a Seahorse XF analyzer in HUVECs. The OCR of cells treated with 20 *μ*M TMP remained higher than those treated with 1 *μ*M Dox (*P* < 0.01, Figure 7(a)). As shown in Figure 7(b), basal respiration before the addition of oligomycin, ATP production after the addition of oligomycin, maximal respiration after the addition of FCCP, and spare respiratory capacity after the addition of rotenone/antimycin A were significantly higher in HUVECs following TMP treatment than Dox treatment. The proton peak was, however, significantly lower with TMP treatment than Dox treatment (*P* < 0.01).

ECAR was used to determine the changes in glycolytic rate in HUVECs. As illustrated in Figure 7(c), the ECAR of TMP-treated cells remained higher than in Dox cells (*P* < 0.01). Basal rates of glycolysis and glycolytic capacity were significantly higher in HUVECs by TMP-treatment following oligomycin injection (*P* < 0.01). In contrast, non-glycolytic acidification increased slightly (Figure 7(d)). These findings suggest that the energetic demand of HUVECs was increased and intracellular acidosis was corrected after TMP treatment due to better maintenance of the mitochondria.

**Figure 8:**
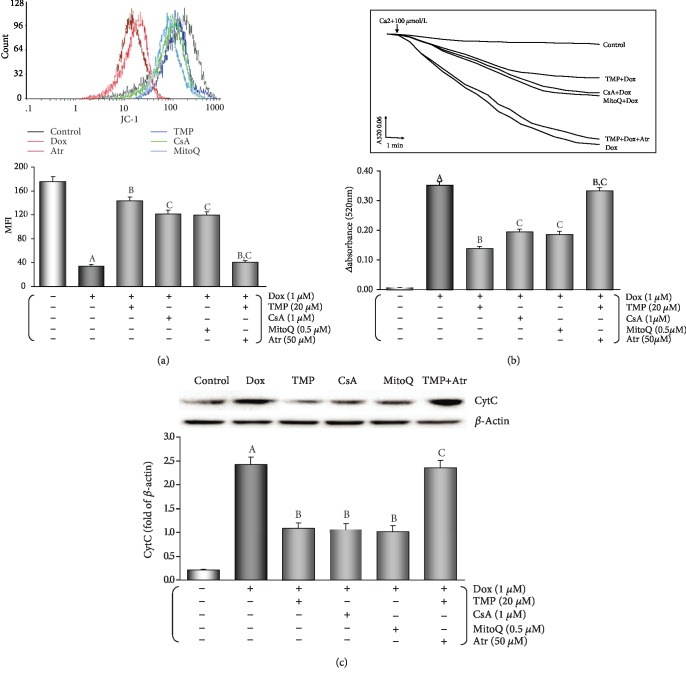
TMP-treated maintains mitochondrial function in HUVECs. (a) MMP levels were evaluated by JC-1. The results of the fluorescence peak on the *x*-axis was used to assess the level of MMP. (b) Ca^2+^-induced swelling of mitochondria was used to determine mPTP opening. The changes in absorbance at 520 nm were detected every 2 minute. The data were accessed by the following equation: *Δ*OD = A520_0min_-A520 _20min_. (c) Western blot analysis and histogram of *cyt C* expression in the cytosol. From left to right, lane 1: control; lane 2: Dox; lane 3: TMP + Dox; lane 4: CsA + Dox; lane 5: MitoQ+Dox; lane 6: TMP + Dox + Atr. Data are presented as the mean ± SEM. for eight individual experiments. a: *P* < 0.01, versus control group; b: *P* < 0.01, versus Dox group; c: *P* < 0.01, versus TMP + Dox group.

Loss of MMP occurs in the early stages of apoptosis. In live cells, JC-1 accumulates in the mitochondrial matrix and only exists in its monomeric form in apoptotic and dead cells because of the loss of MMP [7]. As shown in Figure 8(a), MMP was kept after TMP-treated because the peak of MMP levels significantly shifted to the right (*P* < 0.01). Similarly, CsA or MitoQ treatment also resulted in a significant increase in MMP (*P* < 0.01), but the addition of Atr, co-incubation, resulted a substantial loss in MMP, because of a shift of the peak of MMP to the left (*P* < 0.01).

mPTP opening is a primary cause in cellular apoptosis. The status of mPTP opening was determined by Ca^2+^-induced swelling of mitochondrial [7].  Figure 8(b) shows that when compared with the Dox group opening of the mPTP was inhibited after TMP-treated (*P* < 0.01). CsA or MitoQ treatment also lead to similar effects; however, with the addition of ATR, the effect of TMP-treated on the opening of mPTP was reversed (*P* < 0.01).

On the release of mitochondrial *cyt C* into the cytosol of HUVECs cells were evaluated using Western blot analysis. As shown in Figure 8(c), Dox injury resulted in a significant accumulation of *cyt C* in the cytosol (*P* < 0.01) and *cyt C* in the cytosol was significantly reduced when cells were treated with TMP, or CsA, or MitoQ (*P* < 0.01), but with the addition of ATR, the effect of TMP-treated was canceled (*P* < 0.01).

## 4. Discussion

Dox is a chemotherapeutic drug to treat, including breast cancer, bladder cancer, Kaposi's sarcoma, lymphoma, and acute lymphocytic leukemia [1]. However, in a dose-dependent manner, Dox could cause the irreversible cardiomyopathy [2, 10–12]. In addition to cardiotoxicity, many kinds of cytotoxicity induced by Dox have gradually attracted great attention, especially endothelial dysfunction [3, 4, 48, 49]. It has been noted that the toxicity of Dox to the myocardium and vascular endothelium is often accompanied by and may even cause and effect each other [3–6]. In this study, we could observe the endotheliotoxicity of Dox on vascular endothelium whether *in vivo* or *in vitro*, whether related enzymatic indexes, endothelial function indexes, cell survival, and apoptotic indexes, or morphological indexes (Figures 2–5).

TMP is a main biologically active ingredient purified from the rhizome of *Ligusticum wallichii* [14]. Recently, people has found abundant TMP in mature vinegar and old vinegar [15]. It had been explored many pharmacologically activities of TMP, such as scavenging free radicals, blocking calcium overload, protecting mitochondria, improving energy metabolism, inhibiting cell apoptosis, inducing cytoprotection and so on [15–17]. Many studies have shown that TMP and its derivatives or compatibility with salvianic acid A/resveratrol possess functional cardioprotection/neuroprotection after myocardial ischemia-reperfusion/ischaemic stroke through interfering with PI3K/Akt/GSK3*β*, PGC1*α*/Nrf2, BDNF/Akt/CREB pathway, up-regulating Nrf-2 and HO-1 expression, maintaining Ca^2+^ homeostasis, and inhibiting inflammatory reaction, *etc.* [18–20, 50–52]. Some researchers have shown that TMP and prescriptions as mentioned above can attenuate injury Dox-induced cardiotoxicity and nephrotoxicity by inhibiting oxidative stress, cell autophagy and apoptosis [53, 54]. TMP and the prescriptions can also reverse multidrug resistance induced by Dox in breast cancer, bladder cancer, hepatocellular carcinoma through regulating the expression and function of P-gp, MRP2, MRP3, MRP5, MRP1, GST, Bcl-2, and TOPO-II, or directly enhance the anti-cancer effect of Dox [55–58]. In the study, TMP reversing the vascular endothelium injury induced by Dox-endothelio- toxicity could be confirmed through reducing LDH and CK activities in serum, maintaining EDD, causing a reduction in caspase-3 activity, apoptotic rate, and histopathological changes of vascular endothelium (*in vivo*, Figures 2 and 3). Furthermore, TMP increased cell viability, decreased LDH and caspase-3 activity, and apoptotic rate (*in vitro*, Figures 4 and 5). These results indicate that TMP could reverse or alleviate the damage of Dox toxicity to vascular endothelium *in vivo* and *in vitro*.

Studies have shown that TMP is a multi-target and multi-mechanism ingredient [14], that regulates signaling pathways and affects the expression and activity of specific proteins is one of its prominent mechanisms [14–17]. Our previous work has also found that TMP could up-regulate 14-3-3*γ* expression, phosphorylate Bad (S112), translocate Bcl-2 to the mitochondria, improve mitochondrial function, and reduce apoptosis ultimately against LPS injury in cardiomyocyte [20]. Coincidentally, in this study, both *in vivo* and *in vitro*, with the protective effects on vascular endothelium, TMP could significantly up-regulate 14-3-3*γ* expression, phosphorylate Bad (S112), and Bcl-2 expression, especially in mitochondria. However, pAD/14-3-3*γ*-shRNA, an adenovirus knocking down intracellular 14-3-3*γ* expression, not only knocked down 14-3-3*γ* expression and inhibited Bad phosphorylation, but also significantly decreased Bcl-2 expression in mitochondria (Figures 3(c)-3(f) and 5(a)-5(d)). Concurrently, the protective effects of TMP on vascular endothelium were lost (Figures 2–5). Interestingly, ABT-737, a specific inhibitor of Bcl-2, could not change the up-regulation of 14-3-3*γ* expression and promote Bad phosphorylation. However, it could cancel TMP's effect of Bcl-2 expression in the mitochondria (Figures 3(c)–3(f) and 5(a)–5(d)) and its protective effects on the vascular endothelium (Figures 2–5). Studies have confirmed that Bcl-2 and Bad usually combine to form a complex in the cytoplasm [59]. When stimulated by certain stimuli, at Ser-112 and/or Ser-136 sites, 14-3-3s phosphorylate Bad, Bcl-2 dissociates from the complex and translocate targeting to mitochondria to exert corresponding physiological effects [60]. Combining with the above results of predecessors and ourselves, we could conclude that TMP can significantly up-regulate 14-3-3*γ* expression. Owing to Dox toxicity, 14-3-3*γ* phosphorylates Bad, releases Bcl-2, and translocates targeting mitochondria, reverses or alleviates the vascular endothelial damage. In other words, TMP against the endotheliotoxicity of Dox depends on the 14-3-3*γ* expression and Bcl-2 activity. In addition, some studies have found TMP induces the phosphorylation of eNOS Ser1177 and protects the myocardium [61], in the study, pAD/14-3-3*γ*-shRNA or ABT-737 could not completely inhibit or cancel the effects of TMP on improving p-eNOS/eNOS ratio and NO content (Figures 2(b), 3(g) and 5(e), 5(f)), or that part of the effect of TMP did not depend entirely on14-3-3*γ* expression and Bcl-2 activity.

A growing evidence demonstrate that in Dox-induced cytotoxicity ROS generation caused by triggers subsequent pathophysiological changes [3–6, 10–12]. Studies have shown that Dox accumulates through the reduction of the redox cycling in complex I of electron transport chain (ETC) in mitochondria, thereby increasing ROS generation [62]. Furthermore, mitochondrial NADH-dependent enzymes reduce Dox to corresponding semiquinone radicals, which undergo redox cycles to form superoxide radicals and hydrogen peroxide [63]. In this study, we found that mice after injected Dox, the antioxidant potential was weakened (Table 1, FRAP), the level of oxidative stress was enhanced (Table 1, MDA content, SOD, CAT, and GSH-Px activities). In HUVECs that were treated by Dox, both intracellular/mitochondrial ROS generation significantly increased, NAD^+^, GSSG, and NAD^+^/NADH increased, NADH, GSH, and GSH/GSSG decreased, MitoQ, a mitochondria-targeted CoQ-10 antioxidant, could completely reverse the above changes (Figure 6), indicating that increased oxidative stress was responsible for HUVECs damage, moreover, it provides an experimental basis for “excessive ROS comes from mitochondria” [62]. This result is consistent with the mainstream literature reports. Subsequently, TMP could significantly reverse the increase of oxidative stress induced by Dox, resulting in the antioxidant potential was enhanced, the level of oxidative stress was weakened (*in vivo*, Table 1), a significant decrease in both intracellular/mitochondrial ROS generation, a significant increase in NADH, GSH, and GSH/GSSG, and a significant decrease in NAD^+^, GSSG, and NAD^+^/NADH (*in vitro*, Figure 6). More interestingly, the above effects of TMP are very similar to those of MitoQ, and CsA, a mPTP closing agent, and can be completely reversed by Atr, a potent mPTP opener (Figure 6). It is generally believed that TMP has a certain antioxidant capacity in the cytoplasm [12, 17]. There is no evidence that TMP can scavenge free radicals in mitochondria like MitoQ. Therefore, it is inappropriate to explain the above effects of TMP by its direct antioxidant capacity. Considering the similarity between TMP and CsA, and the reversal of the above effects of TMP after opening mPTP with Atr, we can use the “ROS-induced ROS release” (RIRR) hypothesis to explain it. The hypothesis [64] suggests that when mitochondrial ROS increases, MMP becomes unstable and mPTP opens continuously, then mitochondria swell and rupture, irreversibly damaging mitochondria. Therefore, ROS are released from its matrix into the cytosol and absorbed rapidly by adjacent normal mitochondria, which induces similar changes in adjacent mitochondria, and cascade-like positive feedback amplification, which ultimately leads to apoptosis [65]. Therefore, mPTP openness plays an important role in RIRR, and ROS is the most important stimulus for mPTP opening [66], which forms a vicious circle. TMP could promote the translocation of Bcl-2 to mitochondria. Like CsA, TMP could close mPTP, stabilize MMP, suppress RIRR mechanism, inhibit ROS burst (Figures 6 and 8), terminate vicious cycle, attenuate ultimately the injury of vascular endothelium induced by Dox.

Mitochondria are multifunctional organelles, and can actively or passively drive cellular dysfunction or demise [29, 67]. Indeed, its structural and functional integrity is fundamental to cellular life [29, 68]. In the study, when TMP helps Bcl-2 to translocate in mitochondria, mitochondrial function improves markedly. These include: increased mitochondrial respiration and glycolytic function (the abilities of oxidative phosphorylation and ATP production), corrected intracellular acidosis, normalized energy metabolism (Figure 7), stable MMP, inhibited mPTP opening, and markedly reduced *cyt C* release into the cytoplasm (Figure 8). Therefore, we can conclude that mitochondria are the ultimate target organelles for TMP to protect vascular endothelium from Dox toxicity.

## 5. Conclusion

**Figure 9:**
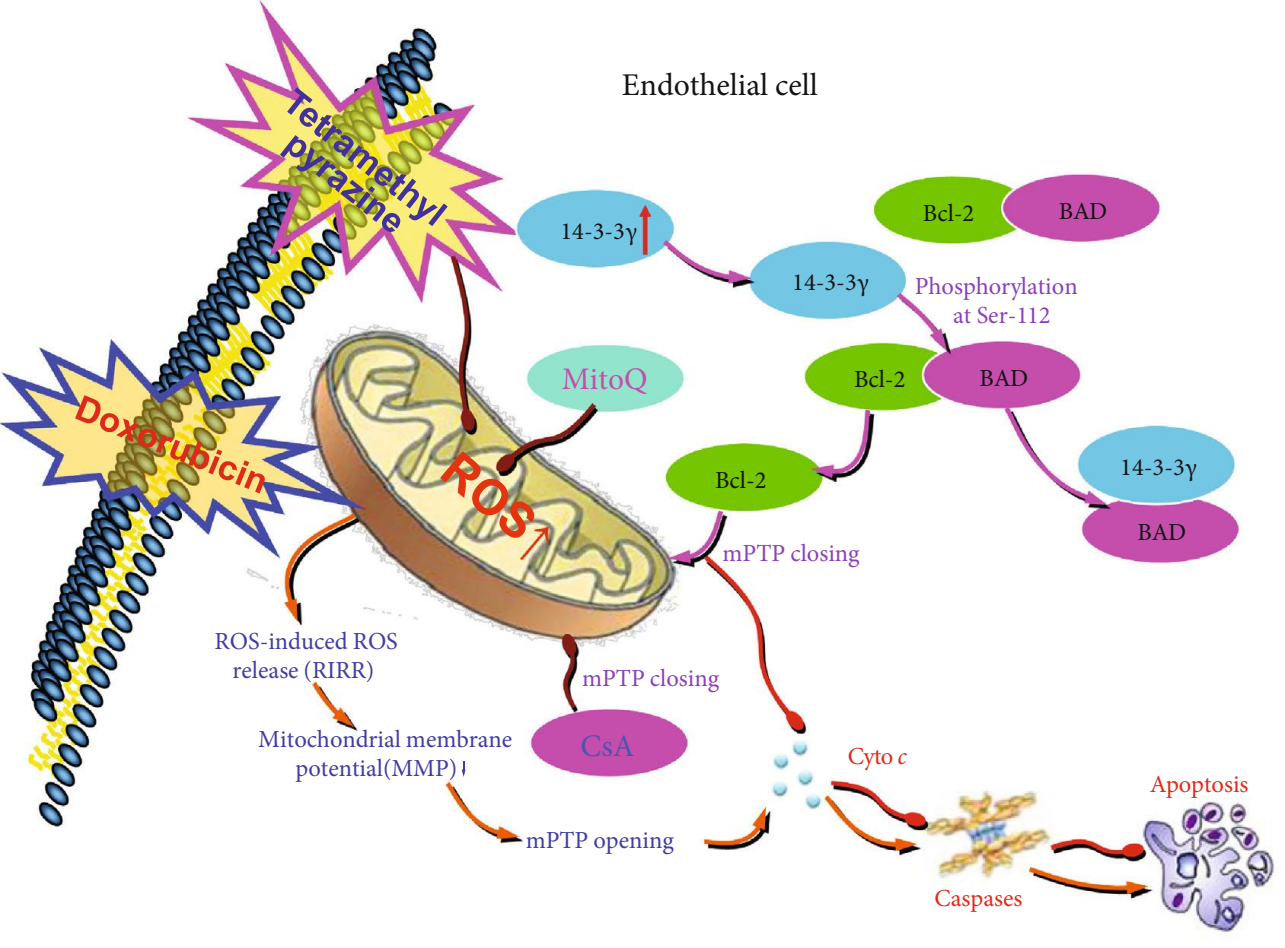
Diagram exhibiting a new mechanism of TMP protects mitochondrial function via 14-3-3*γ*/Bcl-2 in Dox-induced endotheliotoxicity. Dox induced excessive mitochondrial ROS generation, activating RIRR mechanism, weakening MMP, opening mPTP, induce ROS burst, leading to mitochondrial dysfunction, which in turn damages vascular endothelium. TMP upregulated 14-3-3*γ* expression of vascular endothelium, promoted the translocation of Bcl-2 into mitochondria, closed mPTP, keeped MMP, inhibited RIRR mechanism, thereby suppressed oxidative stress, improved mitochondrial function, and alleviated Dox-induced endotheliotoxicity.

In summary, Dox-induced excessive mitochondrial ROS generation, activating RIRR mechanism, weakening MMP, opening mPTP, inducing ROS burst, leading to mitochondrial dysfunction, which in turn damages vascular endothelium. TMP upregulated 14-3-3*γ* expression of vascular endothelium, promoted the translocation of Bcl-2 into mitochondria, closed mPTP, kept MMP, inhibited RIRR mechanism, suppressed oxidative stress, thereby improved mitochondrial function, and alleviated Dox-induced endotheliotoxicity (Figure 9).

## Figures and Tables

**Table 1:** TMP preserved the ferric reducing antioxidant power, the activities of antioxidant enzymes and reduced the levels of lipid peroxidation in the mice's serum against Dox's endotheliotoxicity.

Groups	FRAP (mmol Fe^2+^/L)	SOD activity (U/g protein)	GSH-Px activity (U/g protein)	CAT activity (U/g protein)	MDA content (nmol/g protein)
Control	4.92 ± 0.21	96.1 ± 5.2	26.3 ± 1.8	17.5 ± 1.1	33.2 ± 2.0
Dox	1.56 ± 0.06 ^a^	22.8 ± 1.9 ^a^	5.2 ± 0.3 ^a^	4.7 ± 0.4 ^a^	151.6 ± 14.1 ^a^
TMP + dox	3.60 ± 0.23 ^b^	66.1 ± 3.9 ^b^	17.1 ± 1.5 ^b^	13.6 ± 1.2 ^b^	55.7 ± 5.1 ^b^
TMP + dox + pAD/14-3-3*γ*-shRNA	1.85 ± 0.07 ^b,c^	27.3 ± 2.4 ^b,c^	8.2 ± 0.5 ^b,c^	7.6 ± 0.6 ^b,c^	115.2 ± 12.2 ^b,c^
TMP + dox + ABT-737	1.95 ± 0.09 ^b,c^	29.2 ± 3.1 ^b,c^	9.1 ± 0.8 ^b,c^	7.9 ± 0.8 ^b,c^	102.5 ± 11.5 ^b,c^

Values were presented as mean ± SEM. for fifteen individual experiments. a: *P* < 0.01, versus control group; b: *P* < 0.01 versus Dox group; c: *P* < 0.01 versus TMP + Dox group.

## Data Availability

The data used to support the findings of this study are included within the article.

## Abbreviations

ACh: Acetylcholine
ANOVA: Analysis of variance
Atr: Atractyloside
AUC: Area under the curve
CAT: Catalase
CK: Creatine kinase
CsA: Ciclosporin a
*cyt C*: Cytochrome C
DCFH-DA: 6-carboxy-2`-7`- Dichlorodihydro-flurescein diacetate
DMEM: dulbecco's modified eagle medium
Dox: Doxorubicin
ECAR: Extracelluar acidification rate
EDD: Endothelium-dependent dilation
EID: Endothelium-independent dilation
eNOS: Endothelial nitric oxide synthase
FBS: Fetal bovine serum
FRAP: Ferric reducing antioxidant power
GSH: Reduced glutathione
GSH-Px: Glutathione peroxidase
GSSG: Oxidized glutathione
H&E: Hematoxylin-eosin staining
HUVECs: Human umbilical vein endothelial cells
JC-1: 5, 5', 6, 6'-tetrachloro-1,1',3,3'-tetraethyl- benzimidazolo carbocyanine iodide
LDH: Lactate dehydrogenase
LPS: Lipopolysaccharide
MDA: Malondialdehyde
MitoQ: Mitoquinone
MMP: Mitochondrial membrane potential
mPTP: Mitochonondria permeability transition pore
NAD^+^: Oxidized nicotinamide adenine dinucleotide
NADH: Reduced nicotinamide adenine dinucleotide
NO: Nitric oxide
OCR: Oxygen consumption rate
PBS: Phosphate buffered saline
PE: Phenylephrine
PSS: Physiologic saline solution
RIRR: ROS-induced ROS release
ROS: Reactive oxygen species
SEM: Standard error of mean
SFN: Sulforaphane
SNP: Sodium nitroprusside
SOD: Superoxide dismutase
TUNEL: Terminal deoxynucleotidyl transferase d UTP nick-end labeling
TMP: Tetramethylpyrazine.

## Supplementary Materials

Supplementary Materials Figure S1: Effects of TMP on vascular reactivity of mice's thoracic aortas (endothelium-independent dilation, EID). (A) Endothelium-independent dilation (EID) of the thoracic aortic strips. (B) Area under of the curve for EID of the thoracic aortic strips. Data are presented as the mean ± SEM. for fifteen individual experiments. a: P<0.01, versus control group; b: P<0.01, versus Dox group; c: P<0.01, versus TMP+Dox group. Figure S2: TMP protects HUVECs against the endotheliotoxicity induced by Dox. TMP-treated significantly increased cell viability and reduced LDH activity (P<0.01) in a concentration-dependent manner. (A) Histogram of the cell viability. (B) Histogram of the LDH activity. Data are presented as the mean ± SEM for eight individual experiments. Data are presented as the mean ± SEM for eight individual experiments. a: P<0.01, versus control group; b: P<0.01, versus prior dosage. Figure S3: Effects of TMP/CsA/MitoQ, or downregulated 14-3-3*γ* expression, or inhibited Bcl-2 activity on the cell viability and LDH activity of normal HUVECs. Cell viability and LDH activity did not change by using MP alone, CsA alone, MitoQ alone, pAD/scrRNAi alone, TMP+pAD/14-3-3*γ*-shRNA, TMP+pAD/scrRNAi, TMP+ABT-737, and TMP+Atr when compared with the control group (P>0.05). However, the cell viability of treatment with pAD/14-3-3*γ*-shRNA alone, ABT-737 alone or Atr alone were lower and the LDH activity was higher compared to that of the control group (P<0.01), indicating that 14-3-3*γ* expression, Bcl-2 activity, and mPTP closing play an important role in maintaining normal cell function, and pAD/scrRNAi as a negative control couldn't affect cell viability and LDH activity. (A) Histogram of the cell viability. (B) Histogram of the LDH activity. Data are presented as the mean ± SEM for eight individual experiments. Data are presented as the mean ± SEM for eight individual experiments. a: P<0.01, versus control group. Figure S4: Effects of downregulated 14-3-3*γ* expression, or inhibited Bcl-2 activity on the cell viability and LDH activity of HUVECs by Dox injury. The cell viability of treatment with pAD/14-3-3*γ*-shRNA+Dox, ABT-737+Dox, and Atr+Dox were lower and the LDH activity was higher compared to that of the Dox group (P<0.01) , however, cell viability and LDH activity did not change when using pAD/scrRNAi+Dox (P>0.05), indicating that treatment with pAD/14-3-3*γ*-shRNA down-regulated the expression of 14-3-3*γ*, or using ABT-737 inhibited Bcl-2, or allowing Atr to open the mPTP, thereby aggravating HUVECs injury, and pAD/scrRNAi as a negative control couldn't affect cell viability and LDH activity. (A) Histogram of the cell viability. (B) Histogram of the LDH activity. Data are presented as the mean ± SEM for eight individual experiments. a: P<0.01, versus control group. Figure S5: Effects of TMP, pAD/14-3-3*γ*-shRNA and pAD/scrRNAi on 14-3-3*γ* expression of normal HUVECs. TMP could significantly up-regulated 14-3-3*γ* expression of normal HUVECs, pAD/14-3-3*γ*-shRNA could selectively silence 14-3-3 expression effectively, and pAD/scrRNAi as a negative control couldn't affect 14-3-3 expression. Data are presented as the mean ± SEM for five individual experiments. a: P<0.01, versus control group, b: P<0.01, versus TMP group Figure S6: Effects of TMP/CsA/MitoQ, or TMP added 50 *μ*M Atr on the cell viability and LDH activity of HUVECs injuried by 1 *μ*M Dox. TMP/CsA/MitoQ with 1 *μ*M Dox co-treat HUVECs, the cell viability increased and LDH activity decreased, however added 50 *μ*M Atr could reverse the related effects of TMP. (A) Histogram of the cell viability. (B) Histogram of the LDH activity. Data are presented as the mean ± SEM for eight individual experiments. a: P<0.01, versus control group; b: P<0.01, versus Dox group; c: P<0.01, versus TMP+Dox group.
