# Supplementary material for: Tetramethylpyrazine Attenuates the Endotheliotoxicity and the Mitochondrial Dysfunction by Doxorubicin via 14-3-3γ/Bcl-2
Source: Oxid Med Cell Longev. 2019 Dec 3;2019:5820415. doi: 10.1155/2019/5820415 (PMC6914960; doi:10.1155/2019/5820415)
Supplement: Supplementary Materials — Figure S1: Effects of TMP on vascular reactivity of mice's thoracic aortas (endothelium-independent dilation, EID). (A) Endothelium-independent dilation (EID) of the thoracic aortic strips. (B) Area under of the curve for EID of the thoracic aortic strips. Data are presented as the mean ± SEM. for fifteen individual experiments. a: P<0.01, versus control group; b: P<0.01, versus Dox group; c: P<0.01, versus TMP+Dox group. Figure S2: TMP protects HUVECs against the endotheliotoxicity induced by Dox. TMP-treated significantly increased cell viability and reduced LDH activity (P<0.01) in a concentration-dependent manner. (A) Histogram of the cell viability. (B) Histogram of the LDH activity. Data are presented as the mean ± SEM for eight individual experiments. Data are presented as the mean ± SEM for eight individual experiments. a: P<0.01, versus control group; b: P<0.01, versus prior dosage. Figure S3: Effects of TMP/CsA/MitoQ, or downregulated 14-3-3γ expression, or inhibited Bcl-2 activity on the cell viability and LDH activity of normal HUVECs. Cell viability and LDH activity did not change by using MP alone, CsA alone, MitoQ alone, pAD/scrRNAi alone, TMP+pAD/14-3-3γ-shRNA, TMP+pAD/scrRNAi, TMP+ABT-737, and TMP+Atr when compared with the control group (P>0.05). However, the cell viability of treatment with pAD/14-3-3γ-shRNA alone, ABT-737 alone or Atr alone were lower and the LDH activity was higher compared to that of the control group (P<0.01), indicating that 14-3-3γ expression, Bcl-2 activity, and mPTP closing play an important role in maintaining normal cell function, and pAD/scrRNAi as a negative control couldn't affect cell viability and LDH activity. (A) Histogram of the cell viability. (B) Histogram of the LDH activity. Data are presented as the mean ± SEM for eight individual experiments. Data are presented as the mean ± SEM for eight individual experiments. a: P<0.01, versus control group. Figure S4: Effects of downregulated 14-3-3γ expression, or inhi [file 5820415.f1.pdf]

Supplementary materials

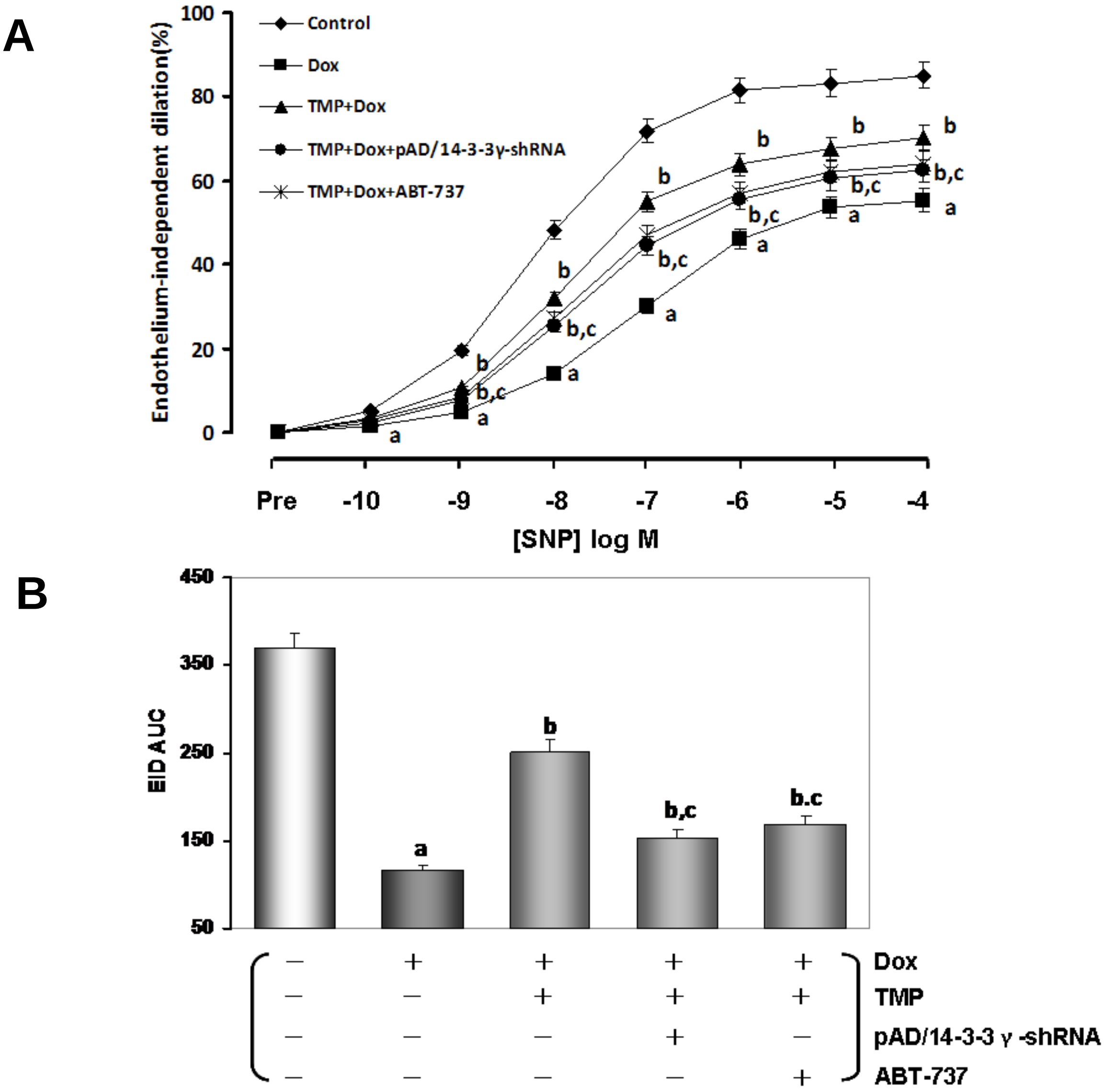

Figure S1: Effects of TMP on vascular reactivity of mice's thoracic aortas (endothe- lium-independent dilation, EID). (A) Endothelium-independent dilation (EID) of the thoracic aortic strips. (B) Area under of the curve for EID of the thoracic aortic strips. Data are presented as the mean ± SEM. for fifteen individual experiments. a: $P<0.01$, versus control group; b: $P<0.01$, versus Dox group; c: $P<0.01$, versus TMP+Dox group.

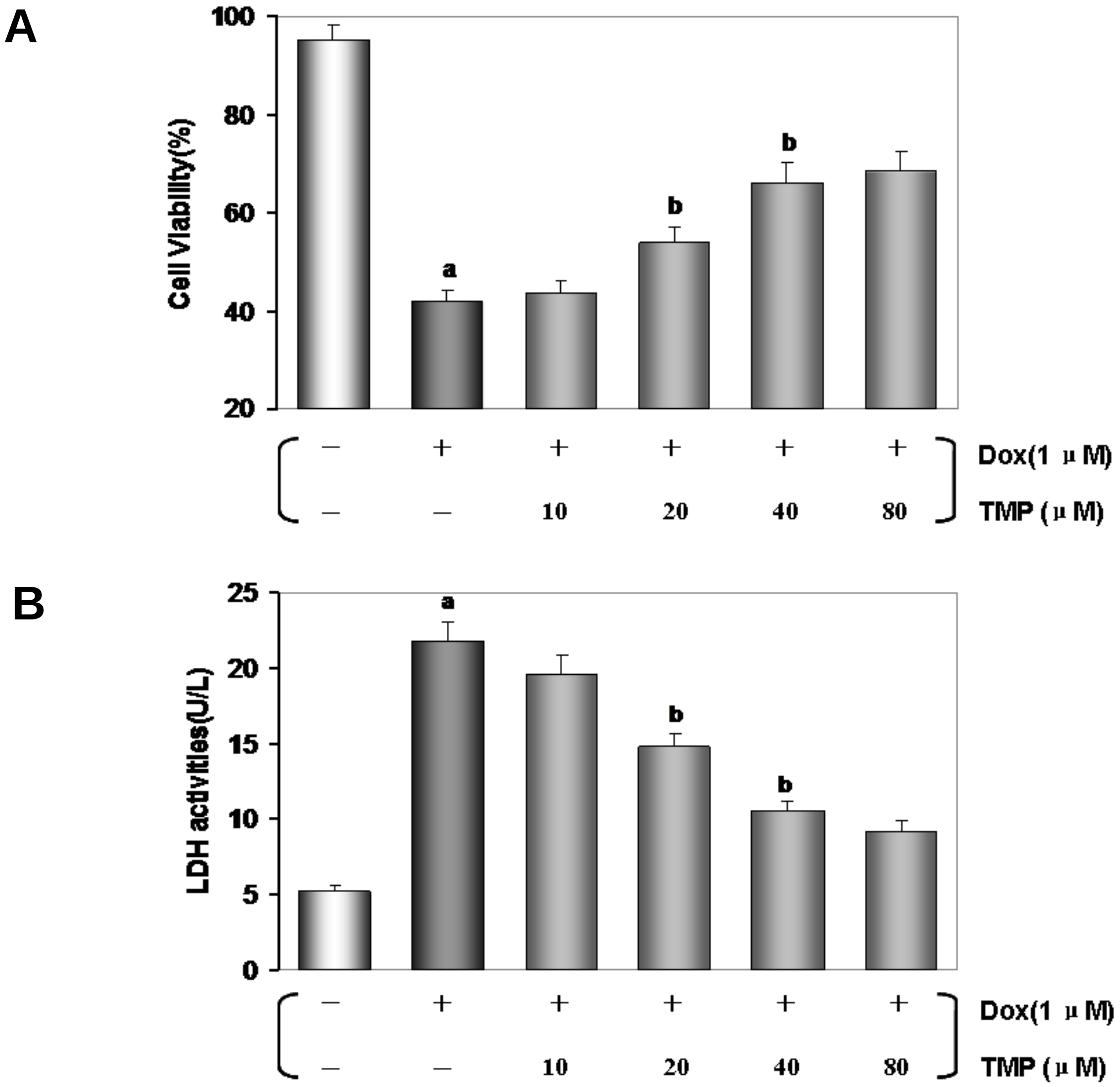

Figure S2: TMP protects HUVECs against the endotheliotoxicity induced by Dox. TMP-treated significantly increased cell viability and reduced LDH activity (*P*<0.01) in a concentration-dependent manner. (A) Histogram of the cell viability. (B) Histogram of the LDH activity. Data are presented as the mean ± SEM for eight individual experiments. Data are presented as the mean ± SEM for eight individual experiments. a: *P*<0.01, versus control group; b: *P*<0.01, versus prior dosage.

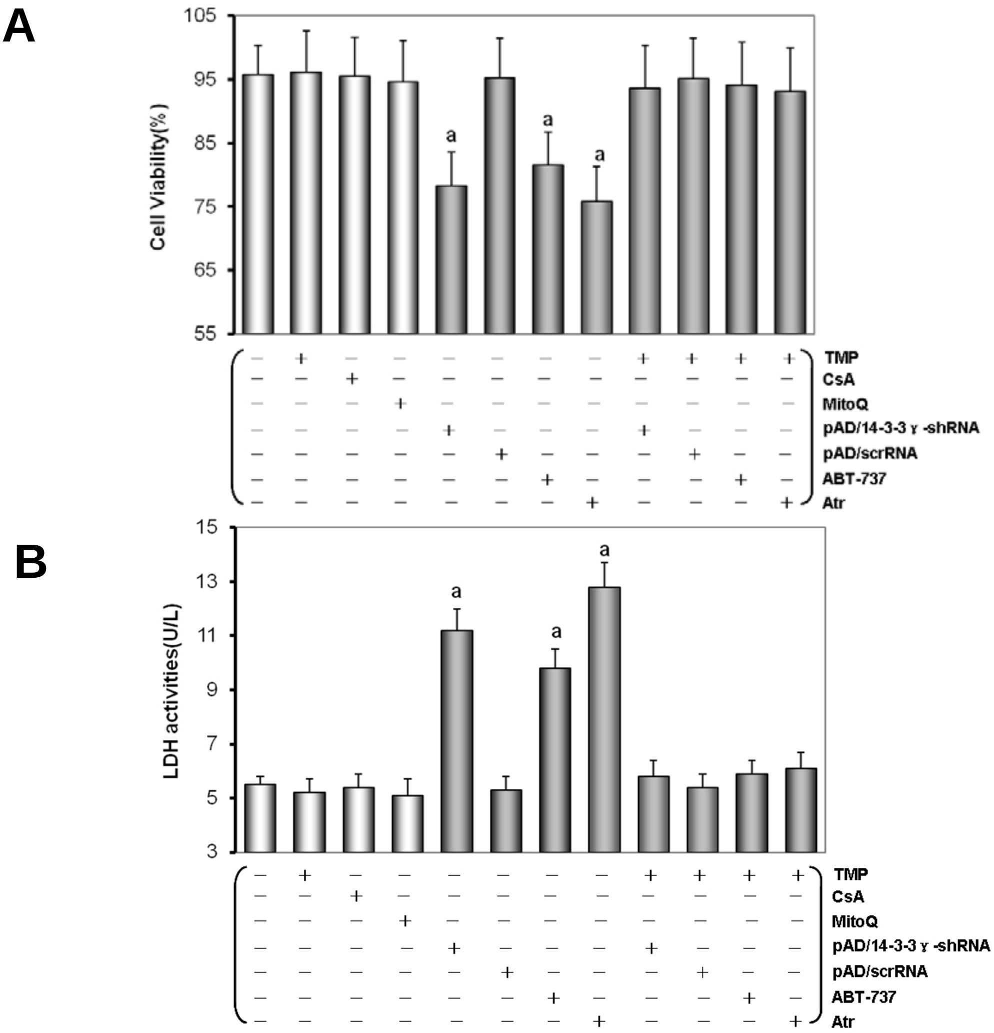

Figure S3: Effects of TMP/CsA/MitoQ, or downregulated 14-3-3γ expression, or inhibited Bcl-2 activity on the cell viability and LDH activity of normal HUVECs. Cell viability and LDH activity did not change by using MP alone, CsA alone, MitoQ alone, pAD/scrRNAi alone, TMP+pAD/14-3-3γ-shRNA, TMP+pAD/scrRNAi, TMP+ ABT-737, and TMP+Atr when compared with the control group (P>0.05). However, the cell viability of treatment with pAD/14-3-3γ-shRNA alone, ABT-737 alone or Atr alone were lower and the LDH activity was higher compared to that of the control group (P<0.01), indicating that 14-3-3γ expression, Bcl-2 activity, and mPTP closing play an important role in maintaining normal cell function, and pAD/scrRNAi as a negative control couldn't affect cell viability and LDH activity. (A) Histogram of the cell viability. (B) Histogram of the LDH activity. Data are presented as the mean ± SEM for eight individual experiments. Data are presented as the mean ± SEM for eight individual experiments. a: P<0.01, versus control group.

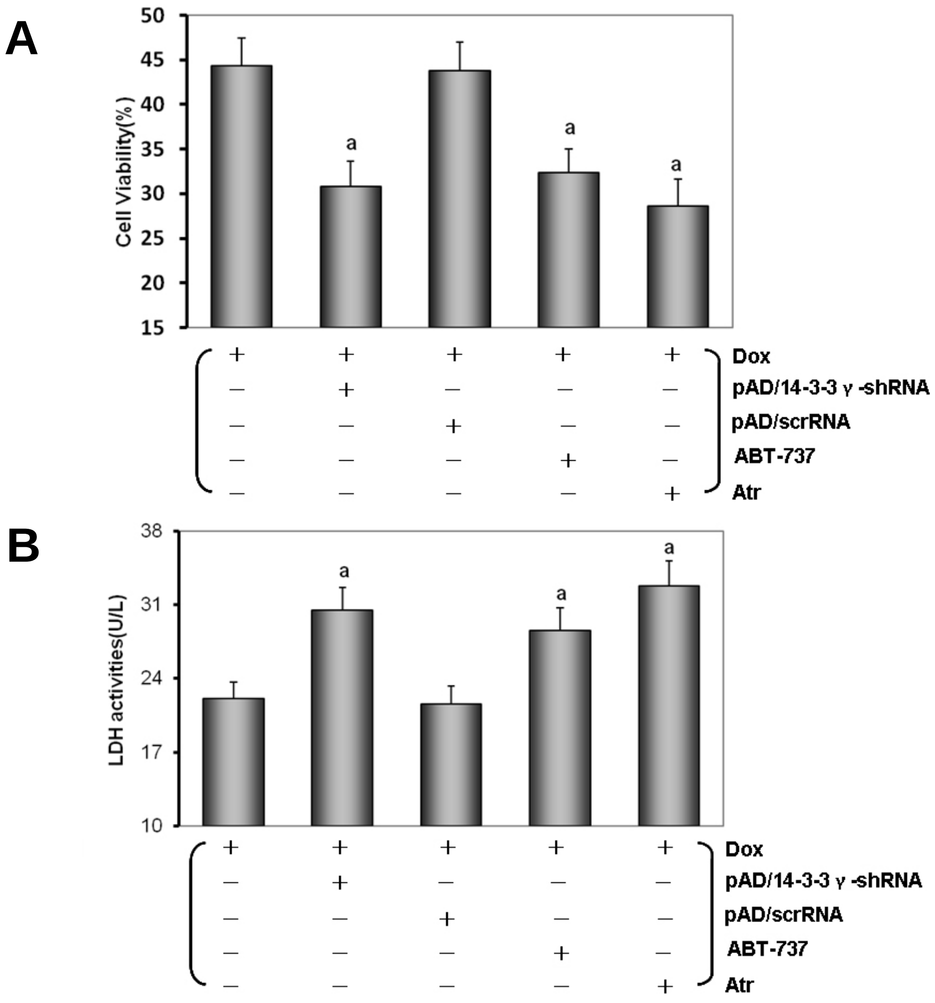

Figure S4: Effects of downregulated 14-3-3γ expression, or inhibited Bcl-2 activity on the cell viability and LDH activity of HUVECs by Dox injury. The cell viability of treatment with pAD/14-3-3γ-shRNA+Dox, ABT-737+Dox, and Atr+Dox were lower and the LDH activity was higher compared to that of the Dox group (*P*<0.01) , however, cell viability and LDH activity did not change when using pAD/scrRNAi+ Dox (*P*>0.05), indicating that treatment with pAD/14-3-3γ-shRNA down-regulated the expression of 14-3-3γ, or using ABT-737 inhibited Bcl-2, or allowing Atr to open the mPTP, thereby aggravating HUVECs injury, and pAD/scrRNAi as a negative control couldn't affect cell viability and LDH activity. (A) Histogram of the cell viability. (B) Histogram of the LDH activity. Data are presented as the mean ± SEM for eight individual experiments. a: *P*<0.01, versus control group.

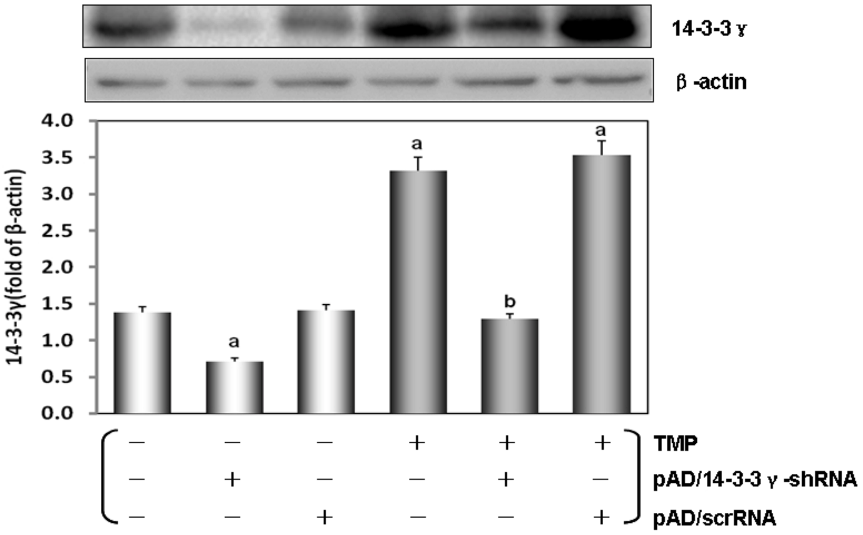

Figure S5: Effects of TMP, pAD/14-3-3γ-shRNA and pAD/scrRNAi on 14-3-3γ expression of normal HUVECs. TMP could significantly up-regulated 14-3-3γ expression of normal HUVECs, pAD/14-3-3γ-shRNA could selectively silence 14-3-3 expression effectively, and pAD/scrRNAi as a negative control couldn't affect 14-3-3 expression. Data are presented as the mean ± SEM for five individual experiments. a: P<0.01, versus control group, b: P<0.01, versus TMP group

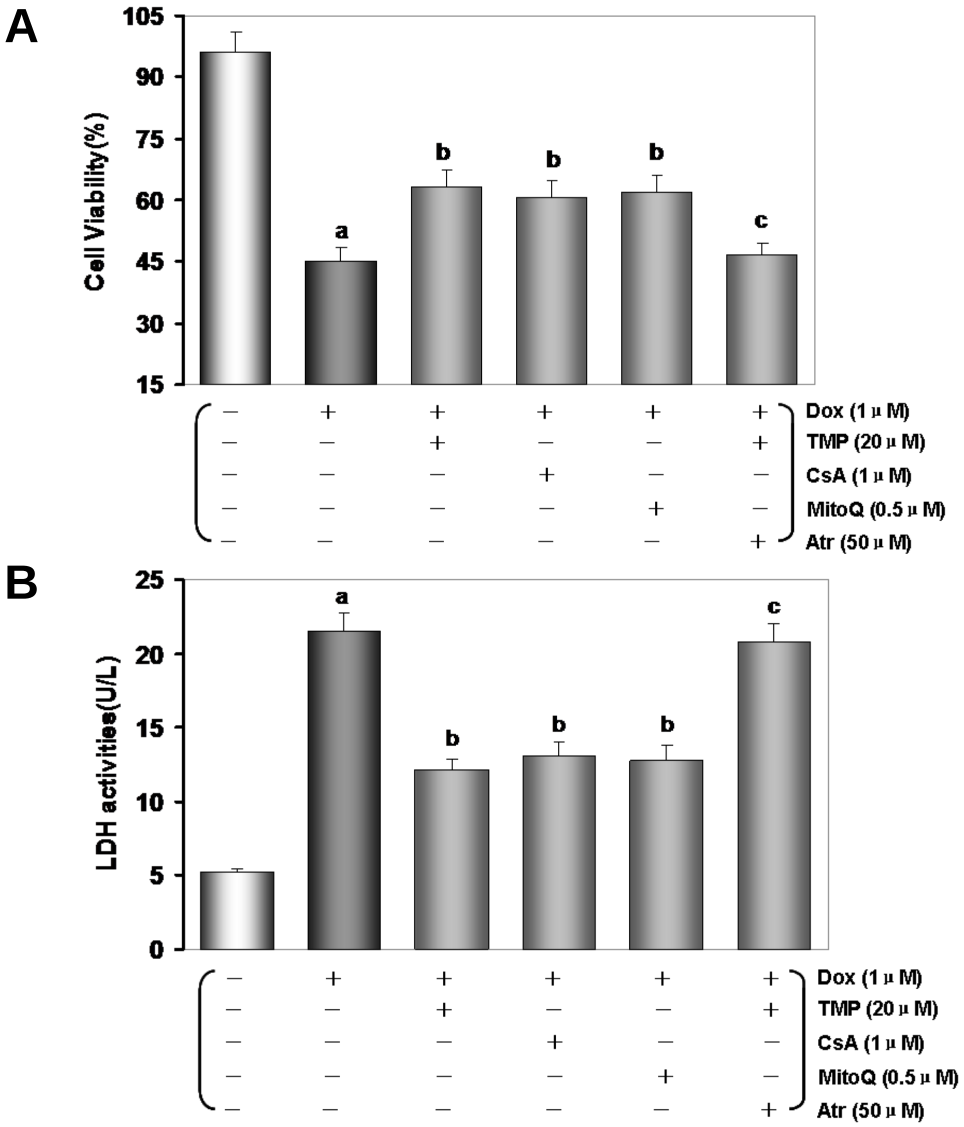

Figure S6: Effects of TMP/CsA/MitoQ, or TMP added 50 μM Atr on the cell viability and LDH activity of HUVECs injuried by 1 μM Dox. TMP/CsA/MitoQ with 1 μM Dox co-treat HUVECs, the cell viability increased and LDH activity decreased, however added 50 μM Atr could reverse the related effects of TMP. (A) Histogram of the cell viability. (B) Histogram of the LDH activity. Data are presented as the mean ± SEM for eight individual experiments. a: *P*<0.01, versus control group; b: *P*<0.01, versus Dox group; c: *P*<0.01, versus TMP+Dox group.
